# VARP binds SNX27 to promote endosomal supercomplex formation on membranes

**DOI:** 10.1126/sciadv.adr9340

**Published:** 2025-02-12

**Authors:** Mintu Chandra, Amy K. Kendall, Marijn G. J. Ford, Lauren P. Jackson

**Affiliations:** ^1^ Department of Biological Sciences, Vanderbilt University, Nashville, TN, USA.; ^2^ Center for Structural Biology, Vanderbilt University, Nashville, TN, USA.; ^3^ Department of Biochemistry, Vanderbilt University, Nashville, TN, USA.; ^4^ Department of Cell Biology, University of Pittsburgh, Pittsburgh, PA, USA.

## Abstract

Endosomes are vital cellular hubs for sorting protein cargoes. Retromer (VPS26/VPS35/VPS29) binds multiple sorting nexin (SNX) proteins on endosomal membranes, but assembly mechanisms of metazoan SNX/Retromer complexes remain elusive. We combine biochemical and biophysical approaches with AlphaFold modeling to identify a previously unidentified direct interaction between SNX27 and VARP. A full biochemical reconstitution system using purified proteins systematically tests how and when coats are recruited to membranes to generate tubules. We demonstrate and measure how specific combinations of Retromer with SNX27, ESCPE-1 (SNX2/SNX6), or both complexes, remodel membranes containing physiological cargo and phospholipids. SNX27, alone and with Retromer, remodels membranes with PI(3)P and PDZbm cargo. ESCPE-1 deforms membranes with bis-phosphoinositides and CI-MPR cargo but surprisingly does not recruit Retromer. VARP co-immunoprecipitates all coat components in cells and is required to reconstitute a proposed endosomal “supercomplex” (SNX27, ESCPE-1, and Retromer) in vitro. These data suggest VARP regulates metazoan endosomal coat assembly to promote cargo sorting out of endosomes.

## Teaser

VARP binds SNX27 to promote the assembly of the SNX27/Retromer/ESCPE-1 endosomal supercomplex on membranes.

## INTRODUCTION

Large multisubunit coat protein complexes initiate distinct trafficking pathways by forming hubs at organellar membranes (*1*). Coats recognize and cluster specific lipid and transmembrane protein cargoes, such as receptors, channels, or enzymes, for packaging into vesicles or tubules. Coats also recruit machinery required to form vesicles or tubules that will ultimately break off from the donor membrane to deliver cargo. Specific coats have traditionally been thought to define different trafficking pathways to ensure that transmembrane cargoes are directed to the correct destination in a timely manner (*2*–*5*). On endosomal membranes, the Retromer heterotrimer composed of VPS26, VPS29, and VPS35 subunits (*6*, *7*) (fig. S1A) plays a role in sorting many cargoes. Retromer can directly bind multiple sorting nexins (SNXs) to form endosomal coat complexes (*4*, *8*–*16*). In yeast, Retromer exists as a stable pentamer composed of the Vps26/Vps35/Vps29 heterotrimer with an SNX heterodimer, Vps5 and Vps17 (*4*, *6*, *14*, *15*, *17*). In metazoans, Retromer has apparently expanded its repertoire to interact with additional SNXs, including orthologous SNX-BAR heterodimers (SNX1/SNX5 and SNX2/SNX6), SNX3, and metazoan-specific SNX27 (*4*, *10*, *12*, *15*, *16*, *18*–*23*). Disruption of Retromer and SNX-mediated trafficking pathways through mutations or protein loss is associated with carboxypeptidase mis-sorting in yeast (*7*) and various human neurological disorders including Alzheimer’s disease, Parkinson’s disease, and Down’s syndrome (*24*, *25*).

SNX proteins belong to a large protein family defined by the presence of a Phox Homology (PX) domain, which recognizes membranes enriched with phosphoinositides (*26*–*28*). Specific SNX proteins have been shown to act as cargo adaptors by trafficking key proteins from endosomes to the plasma membrane or to the trans-Golgi complex (*8*, *28*, *29*). A subset of SNX family members has been shown to deform membranes (*20*, *30*–*33*). BAR domains (Bin/Amphiphysin/Rvs) found in SNX1 and SNX2 have previously been shown to form homodimers (*30*, *33*) to drive or stabilize membrane curvature through a scaffolding mechanism (*18*, *34*, *35*). In some cases, BAR domains can deform membranes through a second mechanism by using an amphipathic helix to insert into one leaflet to generate asymmetry and drive membrane curvature (*30*, *32*, *36*, *37*).

In mammals, Retromer is thought to associate with specific SNX-BAR heterodimers (SNX1/SNX5 or SNX2/SNX6) to retrieve cargo from endosomes back to the *trans*-Golgi network (*18*, *22*, *31*, *32*, *34*) in a pathway analogous to the yeast pentamer. In yeast, the Vps5/Vps17 SNX-BAR heterodimer is proposed to be the functional homolog of the mammalian SNX1/5 or SNX2/6 complexes (*8*, *35*). SNX1/2 and SNX5/6 arose from gene duplication events (*11*, *32*, *38*, *39*), and the purpose of retaining two heterodimer complexes in metazoan cells remains unclear. More recently, SNX1/SNX5 or SNX2/SNX6 heterodimers have been proposed to form the Endosomal SNX-BAR Sorting Complex for Promoting Exit 1 (ESCPE-1) complex (*22*, *33*–*35*). In this model, SNX1 or SNX2 is proposed to act in membrane deformation, while SNX5 or SNX6 further contributes to curvature and recognizes specific motifs found in cargo, including CI-MPR (*22*, *34*, *40*) (fig. S1B). Another protein, SNX3, is conserved from yeast to humans and implicated in sorting distinct cargoes including DMT1-II and Wntless (*41*). SNX3 lacks a BAR domain or any other module known to affect membrane bending. In recent years, pioneering structural studies revealed how some SNX proteins assemble with Retromer on membranes using cryo–electron tomography (*19*, *20*, *41*–*45*). These studies demonstrated tubular structures with Retromer forming V-shaped arches on top of various SNX proteins (yeast Vps5 homodimer, yeast SNX3, or mammalian SNX3) (*20*, *42*, *46*), although these structures use truncated SNX proteins lacking N termini for technical reasons.

The final SNX protein implicated in Retromer-mediated sorting is SNX27, which is unique to metazoans and required for recycling hundreds of transmembrane protein receptors. SNX27 (fig. S1C) has a different domain architecture as compared to SNX-BARs or SNX3. The SNX27 N-terminal PDZ (postsynaptic density 95/discs large/zonula occludens-1) domain binds transmembrane proteins with PDZ binding motifs (PDZbm’s) having the sequence T/S-X-Φ, and PDZbm cargo binding is enhanced by the Retromer VPS26 subunit. The central PX domain enables membrane recruitment through its affinity for dipalmitoyl-phosphatidylinositol-3-phosphate [PI(3)*P*]. The C-terminal FERM (band 4.1/ezrin/radixin/moesin) domain is an interaction module proposed to undertake multiple functions. The FERM domain directly binds short motifs found in SNX1 and SNX2 N termini (*22*, *40*, *47*), as well as small Ras guanosine triphosphatases (*48*, *49*) and transmembrane proteins with NPxY motifs (*12*, *26*, *27*, *40*, *48*–*53*). In metazoans, SNX27/Retromer is proposed to recycle specific cargoes from endosomes to the plasma membrane (*8*, *15*, *22*, *50*, *54*, *55*). SNX27/Retromer has been biochemically and functionally linked to SNX-BARs through binding the N termini of SNX1 and SNX2 (*22*, *40*, *47*). Overall, current models in metazoans suggest that different combinations of SNX proteins bind Retromer to promote either retrieval or recycling of specific cargoes. However, there are currently no published data to demonstrate whether SNX27/Retromer can deform membranes as yeast SNX-BAR/Retromer or SNX3/Retromer complexes can.

In addition to SNX proteins, metazoan Retromer interacts with multiple accessory proteins that regulate its role in endosomal trafficking. Important examples include VARP (VPS9 domain ankyrin repeat protein; also known as ANKRD27; fig. S1D), Rab7, TBC1D5, and the WASH complex subunit FAM21 (*8*, *56*–*62*). Among these proteins, VARP has emerged as a key player in regulating late endosomal dynamics (*54*, *61*–*65*). VARP is a multidomain protein with a VPS9 domain and two ankyrin repeat domains serving as protein-protein interaction modules. VARP functions as a Rab32/38 effector, and it displays guanine nucleotide exchange factor activity toward Rab21. VARP directly binds VAMP7, an R-SNARE involved in endocytic and secretory pathways (*54*, *61*–*65*). VARP recruitment to endosomal membranes relies on binding to VPS29 using two conserved cysteine-rich motifs located adjacent to the two ankyrin repeat domains (*54*, *61*–*65*). A mass spectrometry–based proteomics study of the SNX27 interactome (*66*) recently revealed VARP as a potential SNX27-interacting partner, but the biochemical basis for direct binding between SNX27 and VARP has not been established.

In this study, we use biochemical and biophysical methods together with AlphaFold Multimer modeling to demonstrate a molecular interaction between the N-terminal folded domain of VARP and the SNX27 PDZ domain via the well-established PDZbm binding pocket. Biochemical pull-down assays confirm that both full-length proteins and individual domains interact, while biolayer interferometry (BLI) establishes a relatively strong trafficking interaction [high nanomolar dissociation constant (*K*_d_)]. Structure-based point mutations generated on the basis of AlphaFold computational structures further define sequence requirements for the interaction. Next, we developed a biochemical reconstitution system using purified proteins to systematically establish which combinations of endosomal coat proteins are recruited to liposomes in the presence of relevant phospholipids and cargo motifs. We paired liposome pelleting assays with negative-stain electron microscopy (EM) to ascertain conditions under which combinations of SNX and Retromer proteins can bind and tubulate membranes. These experiments demonstrate for the first time, to our knowledge, how metazoan SNX27 on its own and together with Retromer can deform and tubulate membranes enriched with PI(3)*P* and PDZ cargo motifs. We further show how an ESCPE-1 complex (represented in this work by the SNX2/SNX6 heterodimer) can deform and tubulate membranes enriched with Folch I and CI-MPR cargo motifs, but it does not recruit Retromer. These two different endosomal coats yield tubules having different physical diameters. We tested whether SNX2/SNX6 can engage SNX27/Retromer to form the proposed endosomal “supercomplex” (*18*, *21*, *22*) and find that supercomplex formation depends on the presence of VARP in the reconstitution system. The VARP N terminus alone is sufficient to promote supercomplex formation in vitro, and structure-guided VARP point mutations abrogate the interaction on liposome membranes. In cultured cells, green fluorescent protein (GFP)–tagged VARP can coimmunoprecipitate all supercomplex components. Together, these results reveal an important role for VARP in regulating endosomal trafficking and advance our understanding of how different endosomal coat complexes generate distinct carriers for efficient cargo sorting out of endosomes.

## RESULTS

### VARP directly binds SNX27 in vitro

**Fig. 1.**
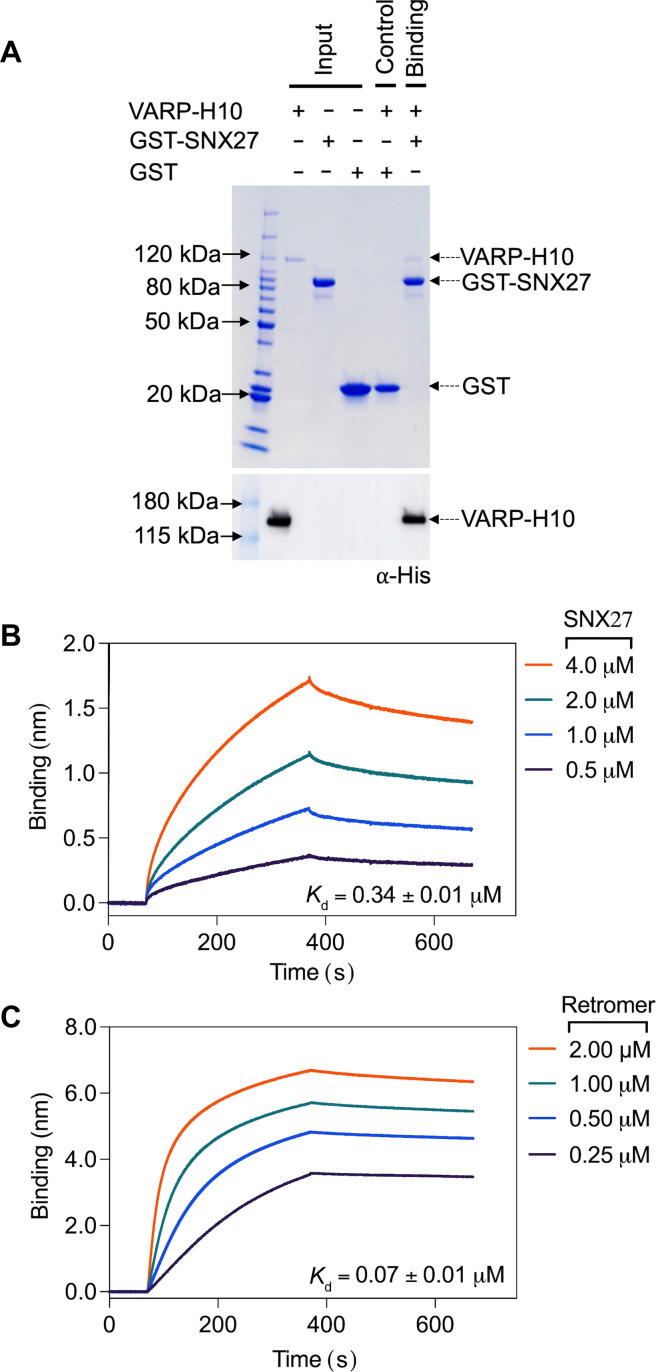
VARP directly binds SNX27 in vitro. (**A**) Coomassie blue–stained SDS-PAGE gel showing GST pull-down experiments with purified GST-SNX27 as bait and VARP-H10 as prey (top panel) and ⍺-His blot (bottom panel). GST-SNX27 is sufficient to pull down VARP FL in vitro. (**B** and **C**) Biophysical data using the BLI Octet system demonstrate that VARP-H10 directly binds SNX27 (B) or Retromer (C) with low micromolar affinity with *K*_d_ values near 1 µM. VARP-H10 was loaded onto a Ni-NTA biosensor, and data were obtained for either SNX27 or Retromer (positive control) at a range of concentrations. Fitted data were plotted in GraphPad Prism, and binding kinetics were calculated using the Octet R8 analysis software package.

Over the past decade, numerous VARP protein binding partners have been identified (fig. S1D), highlighting their diverse roles in Retromer-mediated endosomal trafficking pathways. The SNX27 interactome has been explored using proteomics approaches (*66*), which suggest that VARP and SNX27 may directly bind each other. We tested whether SNX27 could directly bind VARP using recombinant purified proteins in pull-down experiments (Fig. 1A). Glutathione *S*-transferase (GST)–tagged full-length SNX27 (SNX27 FL; GST-SNX27) was used as bait and full-length VARP (VARP FL) with a C-terminal 10×His tag (VARP-H10) as prey. For these experiments, we expressed and purified full-length human VARP in a mammalian expression system (see Materials and Methods). GST-SNX27 efficiently pulls down VARP-H10 (Fig. 1A); the interaction is detected on a Coomassie-stained gel and verified using an antibody against the 10×His tag on VARP. The interaction between VARP FL and SNX27 proteins was further quantified using BLI. A robust dose-dependent increase in the binding between SNX27 and VARP was observed; the calculated average binding affinity (*K*_d_) is submicromolar (0.34 ± 0.01 μM) (Fig. 1B and table S1) with 1:1 stoichiometry (see Materials and Methods). As a positive control, we also measured VARP FL with Retromer (Fig. 1C) at a range of concentrations. These data reveal a nanomolar binding affinity (*K*_d_ = 0.07 ± 0.01 μM) (Fig. 1C and table S1) and 1:2 stoichiometry between one VARP and two Retromer complexes (see Materials and Methods), in line with published data (*63*).

### The folded VARP N-terminal domain directly binds the SNX27 PDZ domain

**Fig. 2.**
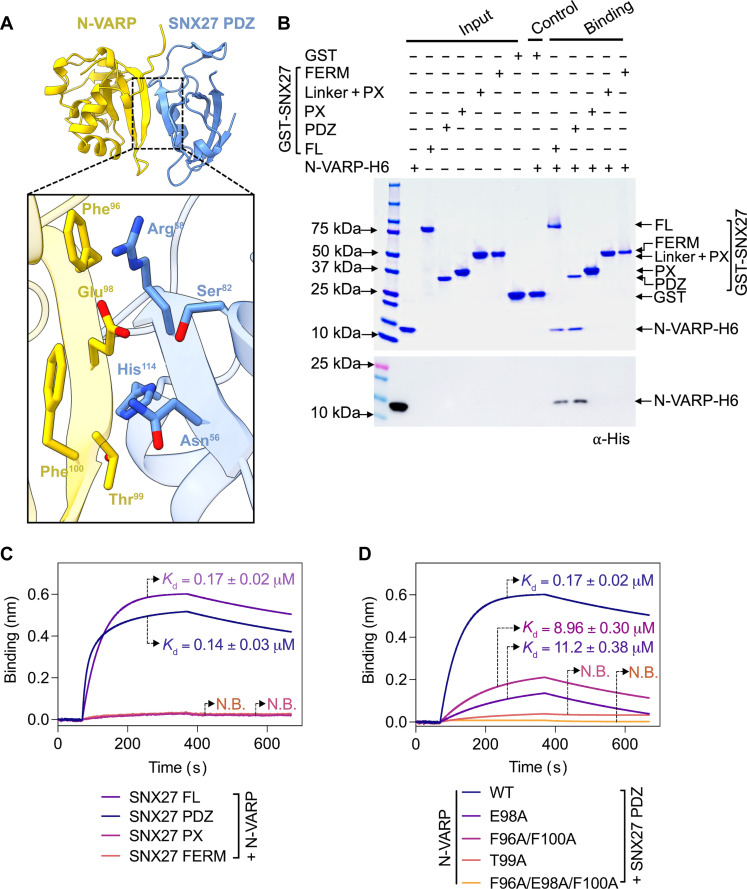
The VARP N-terminal domain directly binds the SNX27 PDZ domain. **(A)** Ribbon diagrams of AlphaFold2.3 Multimer complex model between N-VARP (gold) and SNX27 PDZ (sky blue). The boxed inset shows interacting side-chain residues on N-VARP (gold sticks) and SNX27 PDZ (blue sticks). **(B)** Pull-down experiments with purified GST-SNX27 fusion proteins. SNX27 FL, PDZ, PX, or FERM domains were used as bait with N-VARP-H6 as prey. A representative SDS-PAGE gel stained with Coomassie blue is shown in the top panel with ⍺-His Western blot shown in the bottom panel. (**C**) Biophysical data using the BLI Octet system reveal a low micromolar affinity between SNX27 and N-VARP. Biotinylated N-VARP was loaded onto a streptavidin biosensor for measurements with SNX27 purified proteins. N.B., no detectable binding. (**D**) Hydrophobic residues on VARP drive binding to SNX27. Biotinylated N-VARP mutants (E98A, T99A, F96A/F100A, and F96A/E98A/F100A) were loaded onto a streptavidin biosensor for measurements with the SNX27 PDZ domain. Fitted data were plotted in GraphPad Prism, and binding kinetics were calculated using the Octet R8 analysis software package.

We next turned to AlphaFold Multimer version 2.3 (AF2.3) to generate computational models for the interaction between SNX27 FL and VARP FL (fig. S2). In line with biochemical (Fig. 1A) and biophysical (Fig. 1B) data, AF2.3 models indicated that the N-terminal globular domain of VARP (N-VARP) specifically engages the SNX27 PDZ domain (Fig. 2A). We next generated models using only the VARP N-terminal and SNX27 PDZ domains. These runs consistently produced highly reproducible models with high-confidence predicted local difference distance test (pLDDT) scores (approaching 90; fig. S3) and interface predicted template modeling (ipTM) scores (close to 0.9), along with low predicted aligned error (PAE) scores (close to 0). These metrics strongly support a binding interface between the SNX27 PDZ domain and N-VARP (Fig. 2A). The PISA server (*67*) was used to independently analyze the predicted interface between SNX27 PDZ and N-VARP. PISA analysis reports a substantial buried surface area (1506.4 Å^2^), which further supports a biological interaction leading to the formation of a stable complex (fig. S4, A and C). We further evaluated AF2.3 model geometry using MolProbity (*68*) (table S2). MolProbity reports favorable rotamers and Ramachandran values as well as low Clashscore. Data quality for the AF2.3 model reported here is in line with experimental data from an experimental x-ray structure of the SNX27 PDZ domain with a PDZbm cargo motif [PDB (Protein Data Bank) ID: 5EM9; table S2].

We next validated AF2.3 models using GST pull-down assays with recombinant purified proteins. Full-length GST-SNX27 or GST-tagged SNX27 domains (PDZ, PX, and FERM) were used as bait and His-tagged N-VARP as prey. Consistent with AF2.3 predictions, N-VARP could pull down both SNX27 FL and the SNX27 PDZ domain, but no detectable binding occurred with SNX27 PX and FERM domains alone acting as baits (Fig. 2B). This interaction was quantified using BLI (Fig. 2C). N-VARP binding to either SNX27 FL or the PDZ domain exhibits very similar *K*_d_ values, further indicating that the PDZ domain mediates the interaction.

All AF2.3 computational models suggest that a short sequence within N-VARP (residues 95 to 101; sequence: LFEETFY) binds the conserved SNX27 PDZbm pocket (figs. S4B and S5A). Comparison of the N-VARP sequence with well-established PDZbm motifs revealed similarities with classical type I PDZbm sequences (D/E^−3^−S/T^−2^−X^−1^−Φ^0^, where Φ represents any hydrophobic residue) (fig. S5B). Structural analysis of AF2.3 models reveals specific residues (Fig. 2A) that mediate the molecular interaction between SNX27 PDZ and N-VARP. VARP Thr^99^ (equivalent to the PDZbm −2 position) sits in close proximity to SNX27 His^114^ in the PDZ domain. VARP Glu^98^ (equivalent to the PDZbm −3 position) is located such that it could form electrostatic and hydrogen bonds with SNX27 Arg^58^ and Asn^56^, as well as a hydrogen bond with SNX27 Ser^80^. VARP Phe^96^ (equivalent to the PDZbm −5 position) and Phe^100^ (equivalent to the PDZbm −1 position) residues sit adjacent to SNX27 Arg^58^ and Asn^56^ residues (Fig. 2A and fig. S5A).

Last, we introduced structure-based point mutations into the VARP N terminus to test the necessity of specific residues. We generated two single mutants (VARP T99A and VARP E98A), a double mutant (VARP F96A/F100A), and a triple mutant (VARP F96A/E98A/F100A) for binding studies with the SNX27 PDZ domain in GST pull-down assays (fig. S5C) and BLI experiments (Fig. 2D). Both the VARP T99A mutant and F96A/E98A/F100A triple mutant exhibited no detectable binding to SNX27, while the E98A and F96A/F100A mutants displayed reduced binding compared to the wild-type N-VARP (Fig. 2D). This suggests that VARP Thr^99^ plays a central role in the formation of the VARP and SNX27 complex, while VARP residues Glu^98^, Phe^96^, and Phe^100^ play important auxiliary roles in establishing contacts with SNX27.

### SNX27/Retromer tubulates membranes enriched with PI(3)*P* lipid and PDZbm cargo

**Fig. 3.**
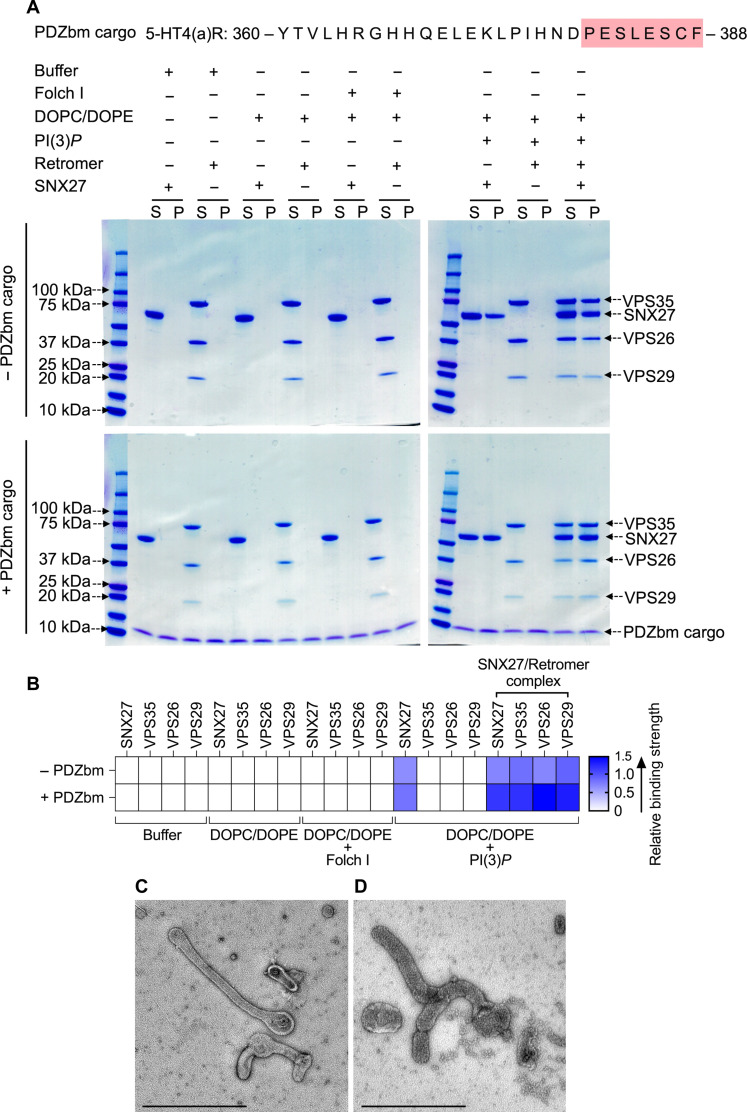
SNX27 and SNX27/Retromer can tubulate membranes in the presence of physiological lipid and cargo composition. (**A**) Liposome pelleting assays demonstrate membrane binding of human SNX27 alone and in the presence of Retromer. Purified recombinant SNX27, Retromer, and SNX27/Retromer complexes were incubated with or without liposomes enriched for PI(3)*P* in the presence or absence of PDZbm cargo from the 5HT4(a)R family (residues 360 to 388; C-terminal PDZbm sequence highlighted in red). Buffer and DOPC/DOPE were used as negative controls to detect nonspecific binding, while Folch I was used to detect broad membrane-binding activity. Samples were subjected to ultracentrifugation followed by SDS-PAGE and Coomassie blue staining of unbound supernatant (S) and bound pellet (P) fractions. (**B**) Protein complex binding to phosphoinositide-enriched membranes visualized by SDS-PAGE was quantified by measuring relative protein band intensities (ImageJ). The enrichment ratio between pellet and supernatant (P/S) was calculated for each protein band in the presence and absence of PDZbm cargo; relative intensity data are plotted as a heatmap. (**C**) Imaging by negative-stain EM reveals robust tubulation of PI(3)*P*-enriched liposomes incubated with SNX27 alone or in the presence of PDZbm cargo. (**D**) Negative-stain EM indicates tubulation of PI(3)*P*-enriched liposomes incubated with SNX27/Retromer in the presence of PDZbm cargo motif from 5HT4(a)R (scale bars, 500 nm).

**Fig. 4.**
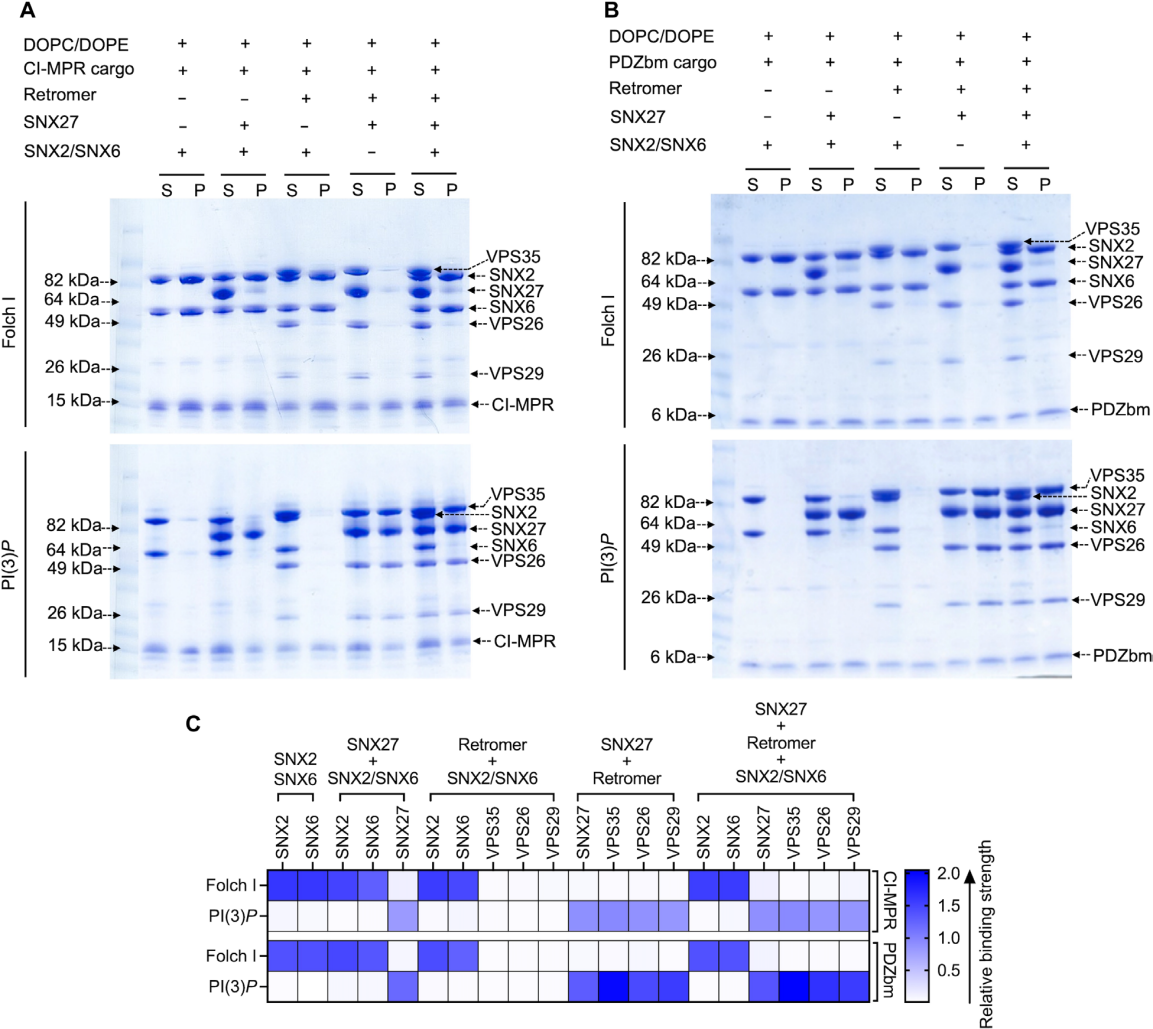
Biochemical reconstitution approaches reveal that SNX2/SNX6 and SNX27/Retromer form different subcomplexes on membranes. Purified recombinant human SNX2/SNX6 (ESCPE-1), SNX27, and Retromer were incubated with (**A**) CI-MPR cargo motif or (**B**) PDZbm cargo motif from 5HT4(a)R in the presence of Folch I–enriched (upper Coomassie gel) or PI(3)*P*-enriched liposomes (lower Coomassie gel). Samples were subjected to ultracentrifugation followed by SDS-PAGE and Coomassie staining of unbound supernatant and bound pellet fractions. (**C**) Protein complex binding to phosphoinositide-enriched membranes visualized by SDS-PAGE was quantified by measuring relative protein band intensities (ImageJ). The enrichment ratio between pellet and supernatant was calculated for each protein complex incubated with either CI-MPR cargo or PDZbm cargo in the presence of Folch I– or PI(3)*P*-enriched liposomes; relative intensity data are plotted as a heatmap. Reconstitution data reveal the specificity of SNX complexes for both phospholipid and cargo composition on liposome membranes. SNX2/SNX6 (ESCPE-1) robustly binds membranes enriched in Folch I and CI-MPR cargo motifs, while SNX27 binds membranes loaded with PI(3)*P* and PDZbm cargo motifs. SNX27 recruits Retromer, while the mammalian SNX2/SNX6 (ESCPE-1) complex does not appear to recruit Retromer in the presence of either cargo or phospholipid.

The next goal was to establish the role of VARP in the context of endosomal coat protein assembly on membranes to establish which combinations of Retromer, SNXs, and VARP can bind and tubulate membranes in vitro*.* Several combinations of SNX proteins with and without Retromer form tubules in vitro, including mammalian SNX1/SNX5 (*33*), yeast and mammalian SNX3/Retromer (*20*), and yeast Vps5/Retromer (*42*). We developed a biochemical reconstitution system using purified mammalian recombinant proteins and cargo motifs (CI-MPR or PDZbm) together with liposomes containing lipid headgroups that mimic physiological compositions [Folch I and PI(3)*P*]. These components were used to conduct liposome pelleting assays (Figs. 3 and 4 and fig. S6) paired with negative-stain EM to visualize and measure diameters of observed tubules.

SNX27/Retromer is widely regarded as an endosomal protein coat on the basis of multiple lines of evidence (*4*, *11*, *12*, *22*, *29*, *43*, *54*), but the ability of this complex to bind membranes and generate tubules has not been directly demonstrated. We first established whether SNX27/Retromer alone can function this way. We conducted liposome pelleting assays with the endosomal head group PI(3)*P* in the presence and absence of PDZbm cargo (Fig. 3A). We generated a purified soluble His-tagged PDZbm cargo protein from the 5-HT4(a)R receptor family because its high affinity toward the SNX27 PDZ domain has been previously established (*50*). The data indicate that SNX27 is specifically recruited to PI(3)*P*-enriched, but not Folch I–enriched, liposomes (Fig. 3A). Retromer requires recruitment by SNX27 to bind PI(3)*P* membranes (Fig. 3, A and B). Last, the presence of PDZbm cargo positively affects the membrane recruitment of both SNX27 and Retromer (Fig. 3A; relative binding strength shown in Fig. 3B). The partial enrichment in liposome pelleting (Fig. 3B) signifies the influence and contribution of the PDZbm cargo, although lipid composition seems to drive membrane binding. As expected, none of the proteins showed binding under various control conditions, including buffer or 1,2-dioleoyl-*sn*-glycero-3-phosphocholine (DOPC)/1,2-dioleoyl-*sn*-glycero-3-phosphoethanolamine (DOPE) alone (Fig. 3 and fig. S7).

We next used negative-stain EM to ascertain whether SNX27/Retromer induces curvature after membrane binding to generate tubules (Fig. 3, C and D). Both SNX27 alone (Fig. 3C) and SNX27/Retromer (Fig. 3D) can drive tubule formation from liposomes enriched with PI(3)*P* and PDZbm cargo, but the tubules have noticeably different diameters. SNX27 tubules exhibit an average diameter of 38.0 ± 5.0 nm (*n* = 20 tubules) (Fig. 3C and table S3), while SNX27/Retromer coats produced wider tubules having an average diameter of 80 ± 6.0 nm (*n* = 50 tubules) (Fig. 3D and table S3). The control experiment using PI(3)*P* liposomes alone did not induce membrane tubulation (fig. S7A). Together, these results indicate how SNX27, with and without Retromer, can induce membrane curvature and tubule formation in vitro*.* The data further highlight how Retromer contributes to membrane remodeling, since its presence induces tubules having a substantially wider diameter.

### SNX2/SNX6 tubulates Folch I membranes displaying CI-MPR cargo

In endosomal coats, SNX-BAR heterodimers have been established as key players in deforming and tubulating membranes (*33*). We reconstituted the mammalian SNX2/SNX6 heterodimer, also known as ESCPE-1 (*33*), to ascertain whether and how it differs from SNX27/Retromer in its ability to bind and tubulate membranes with distinct compositions. SNX2/SNX6 specifically binds liposomes enriched with Folch I, emphasizing specificity for bis-phosphoinositides (PtdIns*P*_2_) over PI(3)*P* liposomes (Fig. 4 and fig. S8A). SNX2/SNX6 exhibited no detectable pelleting in control buffer, DOPC/DOPE, or to PI(3)*P* membranes (fig. S8, A and B). Negative-stain EM revealed that SNX2/SNX6 induces membrane tubulation of Folch I liposomes with an average tubule diameter measuring 55.0 ± 6.0 nm (*n* = 50 tubules) (fig. S8C and table S3). Control liposomes with Folch I alone did not induce tubulation (fig. S7B). Incorporating CI-MPR cargo into the SNX2/SNX6 and Folch I liposome mixture enhanced membrane binding (fig. S8, A and B) and generated tubules with an average diameter of 53.0 ± 5.0 nm (*n* = 50 tubules) (fig. S8D and table S3). Notably, the tubule “run length” increased approximately two- to threefold (fig. S8D) when CI-MPR cargo was present, highlighting its influence on SNX2/SNX6 tubule formation.

### Biochemical reconstitution approaches reveal that SNX2/SNX6 and SNX27/Retromer form distinct subcomplexes having different tubule diameters

We next paired liposome pelleting assays with negative-stain EM to assess which combinations of SNX2/SNX6, SNX27, and Retromer bind and tubulate membranes containing the phospholipid and cargo compositions established independently for SNX27/Retromer and SNX2/SNX6 (previous sections). One notable result was the inability of SNX2/SNX6 to recruit Retromer to Folch I membranes in the presence of CI-MPR cargo; only SNX2/SNX6 was observed in the pellet fraction [Fig. 4, A (upper left Coomassie gel) and C (top row in the heatmap)]. We also tested Retromer recruitment in the presence of PDZbm cargo on Folch I membranes, since SNX2/SNX6 could presumably access this cargo in the context of an assembled supercomplex. However, Retromer is not observed in the pellet fraction [Fig. 4, B (upper right Coomassie gel) and C (third row in the heatmap)]. On PI(3)*P* liposomes, neither SNX2/SNX6 nor Retromer was detected in the pellet fraction [Fig. 4, A and B (lower Coomassie gel) and C (second and fourth rows in heatmap)]. Together, these data indicate the specificity of SNX2/SNX6 for Folch I membranes and support published models, indicating that SNX2/SNX6 alone may function as an independent coat.

Next, we assessed the influence of different subcomplexes on tubule morphology using negative-stain EM. When Retromer was combined with SNX2/SNX6 in the presence of Folch I and CI-MPR cargo, there is a notable decrease in tubulation efficiency (fig. S9A, panel III), even though SNX2/SNX6 is robustly recruited to these membranes in pelleting assays (Fig. 4A). SNX2/SNX6 alone consistently formed elongated tubules (fig. S9A, panel I), but adding Retromer with SNX2/SNX6 resulted only rarely in tubule formation with an average diameter of ~50 ± 5.0 nm (*n* = 5 tubules) (fig. S9A, panel III, and table S3). In line with liposome pelleting data, we rarely detect tubules with SNX2/SNX6 alone or in combination with Retromer on PI(3)*P* liposomes (fig. S9B, panels I and III, and table S3). Notably, control experiments revealed no detectable binding of Retromer alone to either Folch I or PI(3)*P* liposomes (fig. S7C). This notable negative result suggests that the mammalian SNX-BAR/Retromer system may have diverged away from its yeast counterpart.

Last, we sought to establish whether SNX2/SNX6, SNX27, and Retromer form an endosomal supercomplex in the presence of cargo and phospholipids. N-terminal extensions of SNX/BAR family members, including SNX1 and SNX2, have been shown to bind the SNX27 FERM module (fig. S1, B and D) (*31*, *38*, *48*). However, we could not detect efficient pelleting of either SNX27 or the SNX27/Retromer complex with SNX2/SNX6 on Folch I liposomes in the presence of either cargo (CI-MPR or PDZbm) [Fig. 4, A and B (upper Coomassie gels) and C (top and third rows in the heatmap)]. PI(3)*P*-enriched membranes, in the presence of both cargoes, exhibited specificity for SNX27 alone and for SNX27/Retromer, with no detectable SNX2/SNX6 observed in pellet fraction [Fig. 4, A and B (lower Coomassie gel) and C (second and fourth rows in the heatmap)]. In summary, these data reveal that SNX2/SNX6 and SNX27/Retromer bind membranes with distinct compositions, with the phospholipid as a major driver of recruitment in this reconstituted in vitro system.

As before, we analyzed negative-stain EM grids containing membrane-assembled complexes. These images reveal that membranes exposed to SNX2/SNX6 and SNX27 generated membrane tubules with an approximate diameter of 58.0 ± 5.5 nm (*n* = 50 tubules) on Folch I (fig. S9A, panel II, and table S3) membranes, while tubules were rarely detected with PI(3)*P* (fig. S9B, panel II, and table S3). Assemblies with SNX2/SNX6 and SNX27/Retromer complexes exhibited tubules with average diameters of 55 ± 4.0 nm (*n* = 50) for Folch I (fig. S9A, panel IV, and table S3) and 53 ± 5.2 nm (*n* = 50) for PI(3)*P* (fig. S9B, panel IV, and table S3). Notably, both varieties of tubules exhibited a close resemblance to those formed by SNX2/SNX6 alone (average diameter, 55.0 ± 6.0 nm; fig. S9A, panel I, and table S3). This similarity further suggests that these tubules may be primarily decorated with the SNX2/SNX6 complex.

### VARP is required to reconstitute the proposed endosomal supercomplex on membranes

**Fig. 5.**
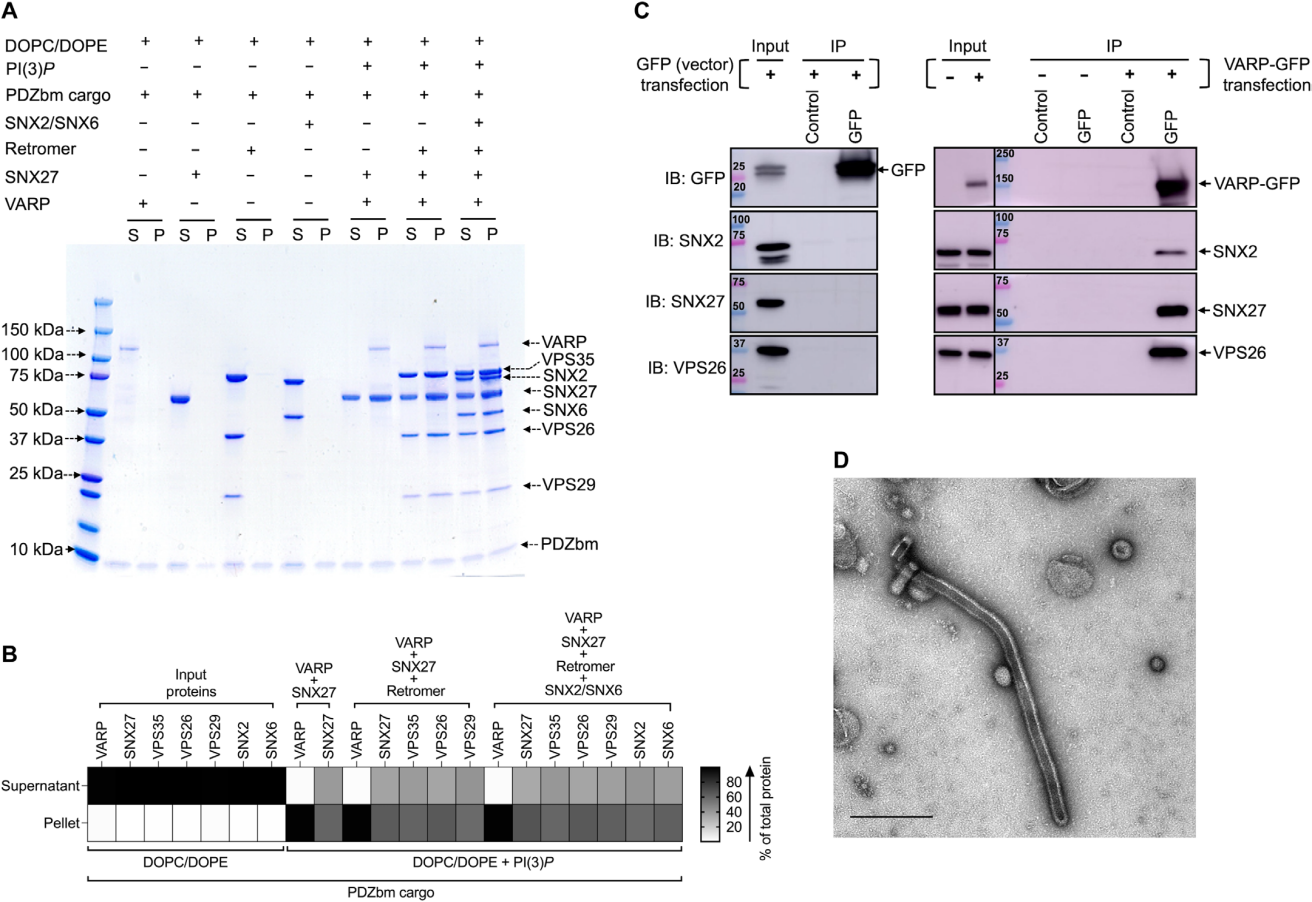
VARP is required in vitro to reconstitute the proposed endosomal “supercomplex.” (**A**) Purified recombinant human SNX27, Retromer, SNX2/SNX6 (ESCPE-1), and VARP were incubated with the PDZbm cargo motif from 5HT4(a)R, either alone or together on PI(3)*P*-enriched liposomes. DOPC/DOPE was used as a negative control. Samples were subjected to ultracentrifugation followed by SDS-PAGE and Coomassie staining of unbound supernatant and bound pellet fractions. In the presence of VARP, all endosomal coat complexes (SNX2/SNX6, SNX27, and Retromer) are recruited to PI(3)*P*-enriched membranes. (**B**) Binding of proteins to PI(3)*P*-enriched membranes in the presence of PDZbm cargo was visualized by SDS-PAGE and quantified using ImageJ to measure the relative intensities of protein bands. The enrichment of proteins in pellet and supernatant fractions was calculated and displayed as a heatmap. The total protein partitioned between the supernatant and the pellet was normalized to 100% and represented in black. Partitioning of proteins between pellet and supernatant fractions is reported with respect to total protein and represented in grayscale (white to black). (**C**) Coimmunoprecipitation experiments using full-length VARP-GFP overexpressed in Expi293 cells. Cell lysates were subjected to GFP trap–based immunoprecipitation (IP) and probed against SNX27, SNX2 (ESCPE-1 component), VPS26 (Retromer subunit), and GFP. Endogenous SNX27, VPS26, and SNX2 coimmunoprecipitate with VARP on GFP-conjugated resin but not under control conditions with unconjugated resin. No detectable bands were observed in untransfected cells or cells transfected with the vector control, pEGFP-N1. The blot is representative of three independent experiments. IB, immunoblotting. (**D**) Representative negative-stain EM image visualizing PI(3)*P* liposomes incubated with the SNX27/Retromer/ESCPE-1/VARP “supercomplex” in the presence of the PDZbm cargo (scale bar, 500 nm). The supercomplex can both assemble [(A) and (C)] and tubulate membranes in the presence of VARP (D).

The finding that SNX2/SNX6 cannot recruit SNX27/Retromer to liposome membranes (Fig. 4, A and B) raises an important question. One likely explanation for failure to observe supercomplex formation is the absence of a protein that allows endosomal subcomplexes to bind each other. The previously unidentified interaction reported here between the VARP N terminus and SNX27 prompted us to test addition of VARP to the biochemical reconstitution. VARP is specifically implicated in SNX27/Retromer recycling to the plasma membrane (*61*), so we conducted pelleting assays with PI(3)*P*-enriched liposomes in the presence of PDZbm cargo. VARP addition yields an approximately stoichiometric complex between SNX2/SNX6 and Retromer in the pellet fraction on PI(3)*P*-enriched membranes [Fig. 5, A (far right lane), and B]. SNX27 appears at slightly higher abundance within this complex, while VARP itself is substoichiometric. These data agree with biophysical data (Fig. 1C) and published data indicating that one VARP binds two Retromer complexes via VPS29 subunits (*63*). In addition, we tested whether endosomal supercomplex proteins interact in the presence of VARP in cultured cells. We conducted coimmunoprecipitation experiments from a modified human embryonic kidney 293 cell line (Expi293) overexpressing VARP-GFP and probed with antibodies against SNX27, VPS26 to test for Retromer, and SNX2 to test for ESCPE-1 (Fig. 5C). These data further support biochemical reconstitution results highlighting robust supercomplex assembly in the presence of VARP. We screened negative-stain EM grids containing the full suite of endosomal proteins (SNX27, Retromer, SNX2/SNX6, and VARP) on liposomes with PI(3)*P* and PDZbm cargo. The observed tubules exhibit an average diameter of 69 ± 3.5 nm (*n* = 50 tubules) (Fig. 5D), which is intermediate in size between tubules formed by SNX27/Retromer (Fig. 3D) and SNX2/SNX6 complexes (fig. S9 and table S3). These data indicate how VARP incorporation induces changes in tubule diameter, which may arise from a change in coat lattice organization (see Discussion).

**Fig. 6.**
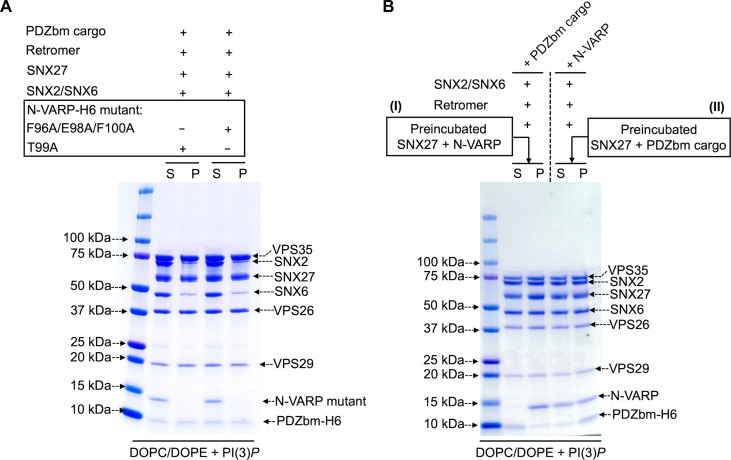
The VARP N terminus is sufficient to recruit an endosomal supercomplex to membranes in vitro. (**A**) Liposome pelleting experiments demonstrate that VARP N-terminal mutants (residues 1 to 117) cannot recruit the endosomal supercomplex to membranes in vitro. Purified proteins of the VARP N-terminal triple mutant (F96A/E98A/F100A) or single mutant (T99A) were incubated with SNX27, SNX2/SNX6, and Retromer in the presence of PDZbm cargo and PI(3)*P*-enriched liposomes. In both experiments, SNX27 and Retromer are recruited, but SNX2/SNX6 exhibits only partial binding to membranes. N-VARP mutants remain in the supernatant fraction. (**B**) A competition experiment demonstrates how binding between N-VARP and SNX27 does not interfere with PDZbm cargo binding and membrane recruitment in the liposome pelleting assays. In experiment (I), full-length purified SNX27 protein was preincubated (see Materials and Methods) with purified N-VARP protein. All endosomal coat protein components are pelleted efficiently in the presence of wild-type N-VARP. SNX27, Retromer, and SNX2/SNX6 are found in the pellet fraction in a ratio similar to that observed for VARP FL (Fig. 5). In experiment (II), full-length purified SNX27 protein was preincubated with purified PDZbm-H6 cargo. All other endosomal proteins were pelleted efficiently in the presence of N-VARP. In experiment (II), there is a greater amount of cargo in the pellet fraction compared to experiment (I). These results together suggest that N-VARP is sufficient to promote endosomal supercomplex formation on membranes and does not inhibit cargo binding or incorporation into the coat.

Last, we tested whether N-VARP alone is required to promote endosomal supercomplex assembly on membranes (Fig. 6). Two approaches were undertaken to test this idea, because computational structural models and biophysical data suggest that N-VARP would compete with PDZbm cargo motif binding. First, structure-based single (T99A) and triple (F96A/E98A/F100A) N-VARP mutants were introduced into liposome pelleting assays (Fig. 6A) in the presence of PDZbm cargo and PI(3)*P.* When either VARP mutant is present, SNX2/SNX6 fails to bind membranes in a manner reminiscent of pelleting assays conducted without VARP (Fig. 4). Neither mutant can reconstitute an approximately stoichiometric supercomplex observed in the presence of VARP FL (Fig. 5A). The second approach involved competition experiments. Isothermal titration calorimetry (ITC) experiments (fig. S10B) further confirm that N-VARP and PDZbm cargo motifs bind the same location on the SNX27 PDZ domain. PDZbm cargo motifs titrated into SNX27 PDZ alone give well-established low micromolar binding affinities (fig. S10B). However, when purified N-VARP is added to SNX27 PDZ in the cell in a 1:1 ratio, the titrated PDZbm cargo peptide exhibits no detectable binding (fig. S10B and table S4). A conceptually similar experiment conducted in the liposome pelleting assay reveals that N-VARP does not impede recruitment of the endosomal supercomplex to membranes enriched with PI(3)*P* and PDZbm cargo (Fig. 6B). Two versions of the competition experiment were designed in the pelleting assay. The first version included a preincubation mixing step between SNX27 and N-VARP (Fig. 6B-I). All components of the endosomal supercomplex were pelleted, but we observe an excess amount of N-VARP and reduced PDZbm cargo in pellet fractions. The second experiment (Fig. 6B-II) included a preincubation step between SNX27 and PDZbm cargo. Here, all protein components in the endosomal supercomplex pelleted efficiently with roughly stoichiometric amounts of N-VARP and PDZbm cargo observed in pellet fractions. We repeated the competitive titration experiments using VARP FL (fig. S11A), which revealed that all protein components pelleted efficiently in the presence of cargo with a substoichiometric amount of VARP FL in the pellet fraction. In contrast, N-VARP is pelleted in roughly stoichiometric amounts (Fig. 6B). These data further support both structural and stoichiometry data. When N-VARP is present, each N-VARP can bind one SNX27 PDZ domain. When VARP FL is present, its stoichiometry decreases, presumably because a single VARP is constrained to bind two VPS29 subunits.

We further tested how cargo affects supercomplex assembly in reconstitution experiments using either purified VARP FL or N-VARP alone in the absence of cargo (fig. S11B). We observed no notable difference between VARP FL pelleting in the presence or absence of cargo (Fig. 5A and fig. S11B). However, N-VARP showed enhanced pelleting onto PI(3)*P* membranes when cargo was absent (fig. S11B). This is consistent with all other data indicating that N-VARP binds the SNX27 PDZ domain, since there will be no competition for the PDZ site in the absence of cargo. Last, we tested explicitly whether PDZbm cargo is incorporated into the endosomal coat rather than being simply associated with Ni-NTA lipid by repeating reconstitution experiments with liposomes omitting Ni-NTA-DGS from the lipid mixture (fig. S11C). We observed PDZbm cargo in the pellet fraction (fig. S11C) at similar levels seen in other experiments (Figs. 3 to 5 and fig. S11A), which demonstrates that PDZbm cargo is present in the complex through binding of SNX27. Together, these data suggest that VARP does not impede PDZbm cargo inclusion in the context of assembled coats and raises many important questions to resolve about the regulatory role of VARP on cellular membranes (see Discussion).

## DISCUSSION

Cellular trafficking pathways rely heavily on interactions between and among multiple protein components to facilitate the sorting and transport of transmembrane protein cargo to their designated destinations. The endosomal system is particularly complex, with multiple protein players interacting across space and time to sort cargoes to different destinations. In this study, we aimed to elucidate how interactions between specific SNX proteins (SNX2, SNX6, and SNX27) and the Retromer complex influence coat formation and membrane tubule morphology. In addition, we established how VARP promotes the formation of the previously proposed endosomal supercomplex composed of SNX27/Retromer and ESCPE-1 through a direct interaction with the SNX27 cargo binding PDZ domain. This biochemical reconstitution approach provides a powerful system to dissect other key protein-protein interactions and to provide testable hypotheses for cell-based experiments.

Published data have demonstrated that VARP is recruited to endosomes through a direct interaction with Retromer (VPS29 subunit) and participates in the SNX27/Retromer recycling pathway that returns the glucose transporter GLUT1 to the plasma membrane (*54*, *61*–*65*). Work presented here provides biochemical and cell-based evidence for an additional and direct interaction between VARP and SNX27/Retromer coats through VARP binding to the SNX27 PDZ domain. Using pull-down assays and BLI, we established that VARP binds both Retromer and SNX27 with high nanomolar binding affinities. The stoichiometry between VARP and Retromer has been established previously (*63*) and here (as a positive control) as one VARP per two Retromers (Fig. 1C). In contrast, BLI data clearly show that VARP interacts with SNX27 with a 1:1 stoichiometry (Fig. 1B).

### The structured VARP N terminus binds SNX27

We established a clear biochemical and biological role for the VARP N terminus through direct binding to the SNX27 PDZ domain. Computational modeling using AF2.3 combined with mutagenesis identified key residues on both VARP and SNX27 PDZ that promote binding. Attempts to crystallize the VARP N terminus have failed in our hands, but multiple versions of AlphaFold (available at https://alphafold.ebi.ac.uk/) as well as models presented here (Fig. 2 and figs. S2 and S3) predict that the N terminus (residues 1 to 117) constitutes a small folded and globular domain. Notably, AF2.3 converges to a predicted model showing how N-VARP uses the sequence LFEETFY to bind the conserved SNX27 PDZ pocket known for its interaction with PDZ binding motifs in transmembrane receptors (*50*). Comparing the N-VARP sequence with C-terminal PDZbm sequences from transmembrane receptors (fig. S5) reveals that the VARP sequence resembles classical type I PDZbm motifs (D/E^−3^−S/T^−2^−X^−1^−Φ^0^, where Φ represents any hydrophobic residue). The function of specific residues found in the VARP sequence would be consistent with prior data from experimental x-ray structures. For example, the Ser/Thr residue at the PDZbm −2 position is crucial for hydrogen bond formation with SNX27 PDZ residue His^114^ and essential for forming the complex between the SNX27 PDZ domain and PDZbm (*50*). Collaborative action of SNX27 residues Arg^58^, Asn^56^, and Ser^80^ provides a binding site for an acidic residue at the PDZbm −3 position. Accommodation of an acidic side chain adjacent to the −5 position by SNX27 Arg^58^ allows the formation of an electrostatic plug. Structural analysis of the AF2.3 model between SNX27 PDZ and N-VARP reveals that N-VARP residues likely maintain similar interactions with the SNX27 PDZ domain, including possible formation of specific hydrogen bonds and ion pairs. We leveraged AF2.3 models to guide mutagenesis studies (Figs. 2D and 6A and fig. S5C) and to independently validate the role of VARP residues in binding SNX27 (Fig. 2 and fig. S5). Overall, combining biochemical and biophysical approaches with computational modeling revealed an important molecular interaction in SNX27/Retromer coat complexes with important implications for coat assembly, cargo recognition, and regulation (discussed below).

### SNX27/Retromer assembles on and remodels membranes in vitro

**Fig. 7.**
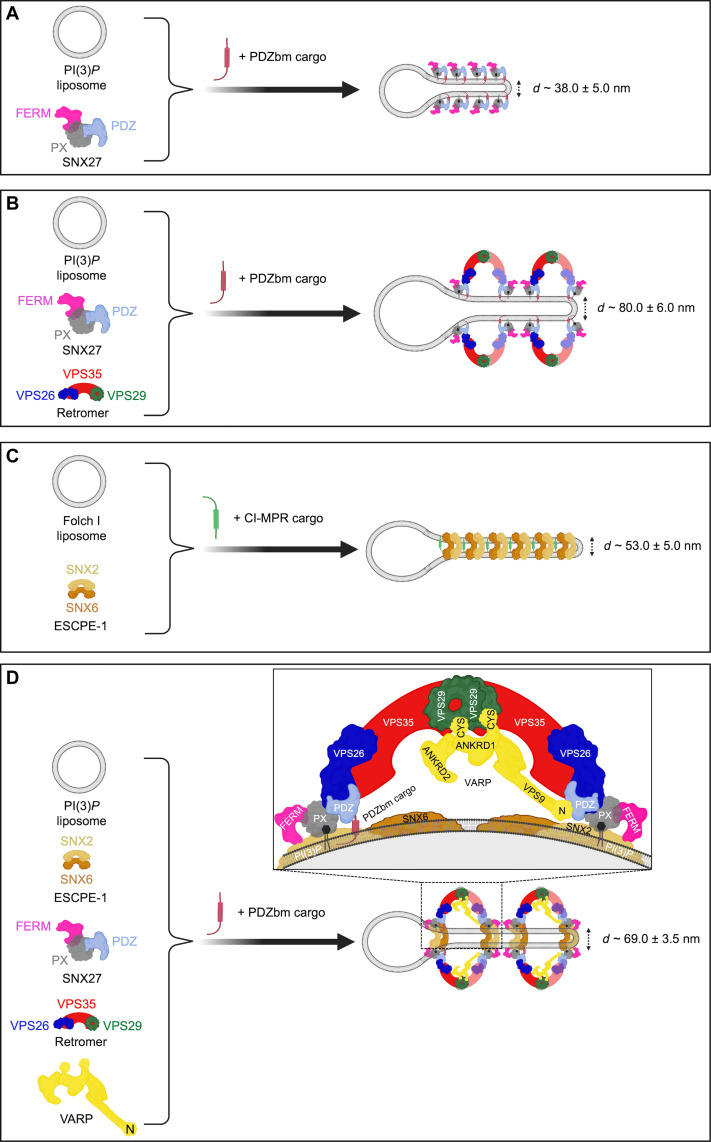
Morphologies of membrane tubules generated by endosomal coat complexes across different lipid and cargo compositions in vitro. Schematic comparison of endosomal coat complex combinations that generate tubules in the presence of physiological lipid and cargo compositions in vitro. Different endosomal coat proteins, either alone or as a complex, produce membrane tubules having different physical diameters as observed in negative-stain EM (table S3). SNX27 alone (**A**) or SNX27/Retromer (**B**) are shown here to generate tubules in the presence of PDZbm cargo and PI(3)*P*. (**C**) SNX2/SNX6 binds a membrane containing Folch I and CI-MPR cargo motifs but does not interact with Retromer under these conditions. (**D**) The assembly of all individual coat components (SNX27, Retromer, and SNX2/SNX6) into the proposed endosomal “supercomplex” occurs on membranes with PI(3)*P* and PDZbm cargo motifs only in the presence of VARP. The stoichiometry quantified using BLI (Fig. 1) suggests that one VARP may bind one SNX27 and two Retromer copies in an arch and promote a conformation allowing the SNX27 FERM domain to bind the flexible SNX2 N terminus (figure created using BioRender.com).

A major goal for this study was to ascertain whether SNX27, alone and with Retromer, could remodel membranes to produce tubules in line with its proposed role as an endosomal coat. Using liposome pelleting assays and negative-stain EM analysis, we demonstrated how SNX27 has inherent membrane-deforming capabilities and can induce tubulation of PI(3)*P*-enriched liposomes having relatively narrow average diameters (38 nm; summary in Fig. 7). Addition of Retromer to the reconstitution results in coated tubules having substantially wider average diameters near 80 nm (Fig. 7 and table S3). These differences highlight how SNX27 and Retromer interact cooperatively to remodel membranes and further support the idea that Retromer acts as a flexible scaffold (*19*). As expected, both the phospholipid composition and cargo presence affect the membrane recruitment of SNX27/Retromer. In the biochemical reconstitution system, phospholipid composition is a major driver for protein recruitment (Figs. 3 and 4), with cargo playing a role in enhancing protein binding. SNX27 exhibits a clear “preference” for binding PI(3)*P* membranes over membranes containing bis-phosphoinositides in Folch I.

The capability of SNX27 alone to deform membranes (Fig. 3C) was unexpected. SNX27 lacks the canonical BAR domain that promotes dimerization and membrane binding in other SNX proteins, including SNX1, SNX2, SNX5, and SNX6. SNX3 also lacks a BAR domain and has been shown to bind Retromer and induce tubulation of PI(3)*P* membranes in the presence of Wntless cargo peptide (*20*, *41*). Together, these results suggest that Retromer-binding SNX proteins have multiple and different mechanisms to bind and shape endosomal membranes. SNX27 interacts with multiple protein- and lipid membrane–associated ligands through its PDZ, PX, and FERM domains. It will be important to understand SNX27 architecture in the context of membrane binding to uncover the underlying mechanism that explains how it can remodel membranes containing PI(3)*P* specifically. In cells, SNX27 works together with Retromer to sort hundreds of important transmembrane receptors linked to neurological health (*12*, *50*, *54*, *55*), so understanding its role is important for both fundamental cell biology and human health.

### ESCPE-1 assembles on Folch I–enriched membranes

We used the SNX2/SNX6 heterodimer to represent ESCPE-1 in this study and to establish how and when ESCPE-1 can engage Retromer and SNX27 in a reconstitution system. These studies revealed an unexpected negative result. For many years, the field has assumed that the mammalian Retromer heterotrimer assembles under some circumstances with SNX1/SNX5 or SNX2/SNX6 to form a pentamer analogous to that observed in budding yeast. Mammalian SNX proteins arose from gene duplication and are orthologs to budding yeast Vps5 and Vps17. Recent data (*22*, *34*, *54*) suggest that specific metazoan SNX-BAR proteins have diverged away from the Retromer heterotrimer, perhaps to form a separate coat called ESCPE-1. ESCPE-1 encompasses different combinations of SNX proteins (*5*, *29*, *31*), and CI-MPR is one important cargo. Here, we focused on SNX2/SNX6 as a model for ESCPE-1, because it was a tractable system for producing high-quality purified proteins. The reconstitution data reproducibly demonstrate how ESCPE-1 robustly binds Folch I–enriched membranes but does not recruit Retromer. SNX27 and ESCPE-1 interact minimally in the absence of VARP despite an established interaction between the SNX2 N terminus and the SNX27 FERM domain (*22*, *40*, *47*). However, we note that there is a small interaction between SNX27 and ESCPE-1 observed in liposome pellet fractions (Fig. 4), while there is essentially no observed Retromer binding. As with SNX27 (previous section), lipid composition is a major driver for ESCPE-1 membrane recruitment. ESCPE-1 robustly binds Folch I–enriched membranes, likely because the SNX2 PX domain exhibits stronger binding to bis-phosphoinositide headgroups including PI(3,4)*P*_2_ and PI(3,5)*P*_2_ (*26*, *69*, *70*). In contrast, the SNX1/SNX5 ESCPE-1 complex (*33*) has been shown to associate with PI(3)*P* but shows minimal interaction with PI(4,5)*P*_2_, PI(3,5)*P*_2_, and PI(3,4)*P*_2_. These data together suggest that different ESCPE-1 complexes composed of distinct SNX heterodimers may recognize membranes having different phospholipid compositions; this could ensure cargo capture under dynamic lipid turnover conditions on endosomal membranes. Our results further demonstrate how ESCPE-1–decorated tubules reproducibly differ in diameter from SNX27 or SNX27/Retromer tubules (table S3). ESCPE-1 forms tubules with average diameters near 53 nm (Fig. 7) in the presence of the CI-MPR cargo peptide. SNX27 cargo–loaded tubules are substantially narrower (38 nm in average diameter; Fig. 7 and table S3), while SNX27/Retromer cargo–loaded tubules are much wider (80 nm in average diameter; Fig. 7 and table S3). CI-MPR is a cargo specific for ESCPE-1 through engaging SNX6 (*33*–*35*). As with SNX27/Retromer, incorporation of a cargo specific to ESCPE-1 somewhat enhances membrane binding and tubulation. The increased (two- to threefold) run length of ESCPE-1 tubules in the presence of CI-MPR is especially notable (fig. S8D), although we were unable to robustly quantify this measurement. Future work, perhaps using focused ion beam milling combined with cryo–electron tomography approaches, in cells characterizing tubule diameter in light of cargo and lipid incorporation will be fascinating to pursue.

### VARP incorporation into endosomal coats has implications for supercomplex assembly, regulation, and cargo sorting

A full biochemical reconstitution system allowed us to test an important unresolved question: Can SNX27, Retromer, and ESCPE-1 form a proposed endosomal supercomplex (*18*, *21*)? ESCPE-1 alone is implicated in retrograde trafficking to the trans-Golgi network, while SNX27/Retromer sorts cargoes to the plasma membrane. But ESCPE-1 has also been observed on recycling tubules (*12*), and reported interactions between the SNX1 and SNX2 flexible N termini and the SNX27 FERM domain further suggest direct binding (*22*, *40*, *47*). Liposome pelleting data revealed that lipid composition and cargo alone were insufficient to promote the formation of the supercomplex (Fig. 4). This result suggested two main possibilities: A supercomplex does not form, or a key regulatory component was missing.

VARP was a notable candidate as the missing component for several reasons. VARP binds Retromer (*61*–*63*), the R-SNARE VAMP7 (*62*), and several Rab proteins (*61*, *64*). VARP uses its two small Cys-rich zinc motifs to bind VPS29, but its location within assembled coats is unclear. VARP has been implicated in GLUT1 recycling in SNX27/Retromer–mediated pathways (*54*). Addition of either full-length purified VARP or the N terminus alone into the reconstitution revealed the first robust biochemical interaction among all components on membranes (Figs. 5 and 6) and suggests that the endosomal supercomplex assembles under certain conditions. These data are insufficient for a robust quantitative approach to establish supercomplex stoichiometry, partly because some proteins (e.g., VPS29) stain less robustly than others. Nevertheless, the interaction between Retromer and ESCPE-1 appears roughly stoichiometric (Fig. 5, A and B), while there is approximately half as much VARP (Fig. 5, A and B). This is in line with biophysical data (Fig. 1) and published work (*63*) suggesting that one VARP engages two Retromers by interacting with VPS29 located at the top of assembled Retromer arches (*42*). In addition, there appears to be an excess of SNX27 relative to other components (Fig. 5, A and B). The assembled supercomplex reproducibly yielded tubules having an average diameter of 69 nm (table S3 and Fig. 7). The different sizes of these tubules from SNX27/Retromer tubules may suggest that VARP regulates coat assembly by altering overall coat composition, architecture, or both. VARP may also introduce asymmetry into arches (discussed further below).

The data presented here prompt an important question regarding cargo recognition and binding in the context of assembled coats. The VARP N terminus engages the well-established cargo binding pocket on the SNX27 PDZ domain (Fig. 2 and figs. S4 and S10) (*50*). This could suggest that cargo and VARP compete for the same binding site on SNX27 PDZ. Calorimetry data here (fig. S10B) and from other labs reveal low micromolar binding affinities (*K*_d_ = 2 to 10 μM) and 1:1 stoichiometry between the SNX27 PDZ domain and multiple PDZ binding motifs (*50*). BLI data (Fig. 1B) reveal a 15 to 30 times higher affinity for the interaction between SNX27 PDZ and VARP. In addition, the full supercomplex is robustly recruited to PI(3)*P* membranes that also contain PDZbm cargo in liposome pelleting assays (Figs. 5 and 6 and fig. S10A). The data suggest that VARP does not hinder cargo binding (at the very least), and this is further supported by liposome pelleting assays using lipids lacking Ni-NTA-DGS, in which PDZbm cargo must bind the SNX27 PDZ domain. Reconstitution experiments in the absence of cargo (fig. S11B), as well as competition experiments (fig. S11A), using both full-length and N-VARP further support a 1:1 stoichiometry between SNX27 and VARP and a 1:2 stoichiometry between Retromer VPS29 and VARP.

Another possibility is that VARP promotes a conformational change in SNX27 that is compatible with both PDZbm cargo inclusion and binding to SNX1 or SNX2 N termini. In cells, there may be a multistep temporal process in which SNX27 is initially recruited to membranes with PI(3)*P* and PDZbm cargo. SNX27 is required to recruit Retromer (Fig. 3), and both proteins harbor binding sites to recruit VARP. Rab’s will also play an important role in establishing membrane identity and ensuring VARP recruitment. One critical role for VARP could be to ensure the packaging of the R-SNARE VAMP7 into the coat for a downstream fusion event. VARP may displace direct PDZbm cargo binding to SNX27 because N-VARP exhibits higher affinity for the PDZ domain. The stoichiometry of the VARP:SNX27 interaction is 1:1, suggesting that VARP may displace only one PDZbm cargo (Fig. 7) while simultaneously incorporating VAMP7, which could bring asymmetry into arches observed in structural studies (Fig. 7D). In liposome assays (Figs. 5 and 6), the presence of full-length or N-VARP alone allows ESCPE-1 to pellet, possibly because the SNX27 FERM adopts a conformation capable of engaging or perhaps releasing the flexible SNX2 N terminus. VARP addition reproducibly produces tubules that differ in diameter from SNX27/Retromer tubules alone (table S3), which further suggest a conformational change in coat architecture.

Overall, the combination of a full biochemical reconstitution system with computational and quantitative biophysical methods provides a powerful suite of tools to test how and when multiple endosomal proteins collaborate to generate tubules in vitro*.* These data can be used for testing hypotheses for how cells can build and regulate tubular transport carriers for efficient cargo sorting out of endosomes. The role of flexible N termini in SNXs and other trafficking proteins in regulating coat architecture is beginning to emerge and will have important implications for eukaryotic cell biology. These ongoing studies have critical implications for human health, since Retromer is considered a viable and important therapeutic target that engages different molecular interfaces when trafficking different cargo proteins.

## MATERIALS AND METHODS

### Molecular biology and cloning

Mammalian Retromer constructs (VPS29, VPS35, and VPS26 subunits) were generated in the labs of D. Owen and B. Collins and have been published previously (*13*, *71*). The original mammalian VARP (ANKRD27) construct was generated in the labs of P. Luzio and D. Owen (*61*, *62*). Human SNX proteins (SNX27, SNX2, and SNX6), N-VARP, and the C-terminal cytosolic tail of PDZbm cargo 5-HT4(a)R (residues 360 to 388; sequence YTVLHRGHHQELEKLPIHNDPESLESCF) and CI-MPR cargo (residues 2347 to 2375; sequence SNVSYKYSKVNKEEETDENETEWLMEEIQ), optimized for *Escherichia coli* expression, were synthesized by the GenScript Corporation (US). All the constructs, except full-length human VARP were cloned either into the pET28A vector with an N-terminal 6×His tag or into the pGEX-4T-2 vector with an N-terminal GST tag for expression and purification. Full-length human VARP was cloned into the mammalian expression vector pcDNA3.4+ with C-terminal 10×His tag for protein purification and was also subcloned into the pEGFP-N1 vector for transient transfection and overexpression in Expi293 cells (Thermo Fisher Scientific, Waltham, MA).

### Antibodies

Antibodies used in this study for Western blot analysis included the following: mouse monoclonal SNX2 (clone 13; 5345661; BD Biosciences) at 1:1000 dilution, SNX27 (ab77799; Abcam) at 1:1000 dilution, horseradish peroxidase (HRP)–conjugated goat anti-GFP (ab6663; Abcam) at 1:4000 dilution, and rabbit polyclonal antibody VPS26A (23892; Abcam) at 1:1000 dilution. The following HRP-conjugated secondary antibodies were used at 1:5000 dilution: Pierce goat anti-rabbit IgG (31460; Thermo Fisher Scientific) and Pierce goat anti-Mouse IgG (31430; Thermo Fisher Scientific). For probing His-tagged prey proteins in GST pull-down assays, an anti-His6-HRP–conjugated primary antibody (ab184607; Abcam) was used.

### Site-directed mutagenesis

A polymerase chain reaction–based method using the Quikchange mutagenesis kit (NEB) was used to generate N-VARP mutants (E98A, T99A, F96A/F100A, and F96A/E98A/F100A) using a plasmid encoding wild-type N-VARP as the template. A pair of oligonucleotide primers containing the desired mutation was used for the polymerase chain reactions (table S5). The template plasmid DNA was linearized by Dpn I digestion before transformation into *E. coli* strain DH5α. Mutations were verified by DNA sequence analysis.

### Recombinant protein expression and purification

All the plasmids used in the current study were transformed into BL21(DE3)/pLysS *E. coli* cells (Promega) and expressed in LB 2xTY broth at 37°C until the absorbance at 600 nm reached 0.8. Cultures were induced with 0.5 mM isopropyl 1-thio-β-d-galactopyranoside and allowed to grow at 20°C overnight, and cells were harvested by centrifugation at 6000*g* for 10 min at 4°C. The cell pellet was resuspended in lysis buffer (20 mM tris, pH 8.0, 200 mM NaCl, 100 U of deoxyribonuclease I, and 2 mM β-mercaptoethanol). The cells were lysed by mechanical disruption at 30 kpsi (206.9 MPa) using a cell disrupter (Constant Systems, UK). The lysate was clarified by centrifugation at 104,350*g* for 30 min at 4°C, and the supernatant was loaded onto a column containing Ni-NTA metal affinity resins (Millipore Sigma, US) for His-tagged proteins. The column was thoroughly washed with lysis buffer containing 100 to 500 mM salt. Last, the protein of interest was eluted with a linear gradient of imidazole (from 100 to 250 mM) in 20 mM tris (pH 8.0) and 200 mM NaCl. Fractions containing the desired protein, as revealed by SDS–polyacrylamide gel electrophoresis (SDS-PAGE), were pooled and dialyzed against gel filtration buffer [20 mM tris, pH 8.0, 100 mM NaCl, and 2 mM dithiothreitol (DTT)]. For GST-tagged constructs, the supernatant from the clarified lysate was loaded onto a column containing glutathione Sepharose 4B resin (Cytiva, US). Again, the column was thoroughly washed with lysis buffer containing 100 to 500 mM salt and, subsequently, either the GST-tagged protein was eluted in 20 mM tris (pH 8.0), 200 mM NaCl, and 20 mM reduced glutathione or the GST tag was cleaved overnight using thrombin (for pGEX4T2 vector-containing constructs) at room temperature, and GST-free protein was eluted using buffer containing 20 mM tris (pH 8.0) and 200 mM NaCl. The eluted affinity purified proteins (His-tagged, GST-tagged, or GST-cleaved) were lastly subjected to size exclusion chromatography using a Superdex-200 16/600 HilLoad column, pre-equilibrated with 20 mM tris (pH 8.0), 100 mM NaCl, and 2 mM DTT, attached to an ÄKTA Pure system (GE Healthcare, US). Fractions containing pure protein, as revealed by SDS-PAGE, were pooled and concentrated using an appropriate cutoff concentrator (Centricon, Millipore Sigma, US) and stored at −80°C.

Mammalian VARP was transiently expressed using the Expi293 Expression System (Thermo Fisher Scientific, Waltham, MA). Cells were grown in 250-ml flasks to a volume of 75 × 10^6^ cells per flask and then transfected with 1.0 μg of plasmid DNA per milliliter of culture using the ExpiFectamine 293 kit (Thermo Fisher Scientific, Waltham, MA). Cells were harvested between 68 and 75 hours posttransfection and frozen at −20°C until use. The frozen cell pellet was resuspended in lysis buffer (20 mM tris, pH 8.5, 500 mM NaCl, 100 U of deoxyribonuclease I, 4 mM MgCl_2_, and 2 mM β-mercaptoethanol). The cells were lysed using a dounce homogenizer and subsequently passed five times through an 18-gauge needle. The lysate was clarified by centrifugation at 104,350*g* for 30 min at 4°C, and the supernatant was loaded onto a column containing Ni-NTA metal affinity resins (Millipore Sigma, US). The subsequent purification steps were carried out analogous to the purification of a His-tagged protein, as described above.

### Phospholipids

DOPC, DOPE, 1,2-dioleoyl-*sn*-glycero-3-phospho-l-serine (DOPS), and 1,2-dioleoyl-*sn*-glycero-3-[(*N*-(5-amino-1-carboxypentyl) iminodiacetic acid) succinyl] (DGS-Ni-NTA) nickel salt were purchased from Avanti Polar Lipids. PI(3)*P* was purchased from Echelon Biosciences, and Folch I (crude brain extract) was purchased from Sigma-Aldrich.

### Liposome preparation

All the phosphoinositides were protonated before usage. Briefly, powdered lipids were resuspended in chloroform (CHCl_3_) and dried under argon. Dried lipids were then left in a desiccator for 1 hour to remove any remaining moisture. Dried lipids were resuspended in a mixture of CHCl_3_:methanol (MeOH):1 M hydrochloric acid in a 2:1:0.01 molar ratio, and lipids were dried once again and allowed to desiccate. Lipids were then resuspended in CHCl_3_:MeOH in a 3:1 ratio and dried once again under argon. Last, dried lipids were resuspended in CHCl_3_ and stored at −20°C.

Folch I liposomes were formulated by mixing DOPC, DOPE, DOPS, and DGS-Ni-NTA in a molar ratio of 42:42:10:3 with Folch I (1 mg/ml). Similarly, liposomes containing PI(3)*P* were prepared by mixing DOPC, DOPE, DOPS, and DGS-Ni-NTA in a molar ratio of 42:42:10:3 with 3 mol % PI(3)*P*. Both types of liposomes were prepared in a buffer containing 20 mM Hepes-KOH (pH 7.5), 200 mM NaCl, and 1 mM tris(2-carboxyethyl) phosphine at a final concentration of 1.0 mg/ml by performing five cycles of freeze-thaw steps followed by extrusion through a 0.4-μm polycarbonate filter. Control liposomes were prepared by combining DOPC and DOPE at a molar ratio of 80:20.

### Liposome pelleting

For liposome pelleting experiments, either Folch I liposome (0.5 mg/ml), PI(3)*P* liposome (0.5 mg/ml), or control DOPC/DOPE liposome (0.5 mg/ml) was used with the individual protein/protein complex sample(s) to a final volume of 100 μl. The following protein concentrations were used for the liposome pelleting experiment: For the liposome pelleting experiments, 2.5 μM Retromer complex, 5 μM SNX2/SNX6 heterodimeric complex (twofold), 10 μM SNX27 (fourfold), and 100 μM cargo adaptors (PDZbm or CI-MPR) (40-fold) were combined. The reaction mixture containing protein(s) and liposome in the presence or absence of cargo adaptors was left at room temperature (25°C) for almost 1 hour to allow for protein-liposome interaction. After incubation, the solution was centrifuged at 50,000*g* for 45 min. Supernatant and pellet fractions were separated, and the pellet was resuspended in a buffer containing 20 mM Hepes-KOH (pH 7.5), 200 mM NaCl, and 1 mM tris(2-carboxyethyl) phosphine. Samples were then collected for analysis separated on a precast 4 to 12% tris-glycine gel (Bio-Rad) and stained with Coomassie. The binding of the protein-phosphoinositide interactions within SDS-PAGE has been further quantified by measuring the protein band intensities in ImageJ (https://imagej.net/ij/). The enrichment of the fraction of pellet and supernatant was calculated for each protein band, both in the presence and absence of relevant cargo, and plotted as a heatmap.

In competitive liposome experiments, SNX27 FL was preincubated with either N-VARP (version I) or PDZbm-H6 cargo (version II) and PI(3)*P* liposomes at room temperature (25°C) for 1 hour. After the preincubation step, purified Retromer and SNX2/SNX6 were added to either N-VARP (version I) or PDZbm cargo (version II) in the preincubation mixture and the reaction was allowed to proceed for an additional 1 hour. After the preincubation step, samples were centrifuged at 50,000*g* for 45 min. The supernatant and pellet fractions were separated, and the pellet was resuspended in buffer containing 20 mM Hepes-KOH (pH 7.5), 200 mM NaCl, and 1 mM tris(2-carboxyethyl) phosphine. The samples were analyzed by SDS-PAGE on a precast 4 to 12% tris-glycine gel (Bio-Rad) and visualized using Coomassie staining.

### Negative-stain EM

For tubulation assays, liposomes [0.5 mg/ml; Folch I or PI(3)*P*] were incubated with either 5 μM of each individual protein (SNX27, Retromer, or SNX2/SNX6) in the presence of their respective cargoes (PDZbm or CI-MPR) or with protein combinations (2.5 μM Retromer, 5 μM SNX2/SNX6, and 10 μM SNX27 along with 100 μM of respective cargoes) for 4 hours at room temperature (25°C). Sample aliquots (10 μl; protein with liposomes or liposomes alone) were adsorbed to glow-discharged 400-mesh carbon-coated copper grids (Electron Microscopy Sciences, EMS, US) and stained with 0.75% uranyl formate and 1% uranyl acetate. The grids were examined on a Tecnai FEI Thermo Fisher Scientific Morgagni 100-kV transmission electron microscope, and images were recorded on a 1k × 1k AMT charge-coupled device camera. Tubule diameters were quantified in ImageJ analysis software (https://imagej.net/ij/) as an average of three measurements along the tubule.

### GST pull-down assays

Full-length GST-tagged SNX27 (1 nmol) was mixed with 1 nmol of full-length His-tagged VARP for 1 hour at 4°C. The protein mixture was then centrifuged at high speed to remove any precipitated proteins. The supernatant was then added to pre-equilibrated (20 mM tris, pH 8.0, 200 mM NaCl, and 1 mM DTT) glutathione Sepharose resin and allowed to mix for an additional 30 min at 4°C. Beads were washed five times in the above buffer supplemented with 0.5% Triton X-100 (Sigma-Aldrich). Bound proteins were analyzed by Coomassie staining and Western blotting of SDS-PAGE gels. For Western blot analysis, His-tagged prey proteins were detected using anti-His6-HRP–conjugated primary antibody (Abcam, ab184607). Uncropped Western blot images are displayed in fig. S12.

### Biolayer interferometry

The kinetics of the protein-protein interactions were determined using BLI from the BLI system (Sartorius Octet BLI Discovery). Protein-protein interactions were observed by immobilizing His-tagged VARP FL (0.05 mg/ml) on a Ni-NTA biosensor or biotinylated N-VARP (0.05 mg/ml) on a streptavidin biosensor. After immobilization, the sensor was washed with buffer containing 10 mM tris (pH 8.0), 150 mM NaCl, and 0.1% bovine serum albumin to prevent nonspecific association. Increasing concentrations of SNX27 FL (0.5, 1.0, 2.0, and 4.0 μM) and Retromer (0.25, 0.5, 1.0, and 2.0 μM) were added to the Ni-NTA biosensor, whereas increasing concentrations of SNX27 FL, PDZ, PX, and FERM domains (0.25, 0.5, 1.0, and 2.0 μM) were added to the streptavidin biosensor. The binding changes (nanometers) were measured in separate experiments performed in triplicate. Proteins were then allowed to disassociate from the probe in the same buffer. The data were processed and plotted using the Octet R8 analysis software package. Data from runs with VARP FL and SNX27 proteins exhibited better fitting with the 1:1 stoichiometric binding model (*R*^2^ = 0.99) compared to the 1:2 binding model (*R*^2^ = 0.87). Similarly, data from runs with VARP FL and Retromer heterotrimer exhibited better fitting with the 1:2 stoichiometric binding model (*R*^2^ = 0.99) compared to the 1:1 binding model (*R*^2^ = 0.92).

### Isothermal titration calorimetry

ITC measurements were conducted on a Nano-ITC instrument (TA Instruments) in buffer consisting of 20 mM Hepes (pH 7.5), 100 mM NaCl, and 2 mM DTT. The PDZbm cargo peptide 5-HT4(a)R-pS^-5^ (phosphorylated at the serine −5 position; commercially synthesized from GenScript) was dissolved in 20 mM Hepes (pH 7.5), 100 mM NaCl, and 2 mM DTT for use in ITC binding experiments. In one experiment, the 5-HT4(a)R-pS^-5^ peptide was titrated with purified SNX27 PDZ domain; in a second experiment, the 5-HT4(a)R-pS^-5^ peptide was titrated with a preincubated mixture of purified SNX27 PDZ and N-VARP domain proteins. In a typical experimental setup, the sample cell was filled with 300 μl of SNX27 PDZ domain protein or a preincubated mixture of SNX27 PDZ and N-VARP proteins. The syringe contained a 50-μl solution of 5-HT4(a)R-pS^-5^ synthetic peptide (residues 1330 to 1336, EpSLESCF). All solutions were degassed before being loaded into the cell. Aliquots (2 μl) of a 0.5 mM peptide solution from the syringe were injected into 25 μM SNX27 PDZ or a preincubated mixture of SNX27 PDZ and N-VARP domain protein solution at 25°C with an interval gap of 3 min and with the syringe rotating at 150 rpm to ensure proper mixing. Data were analyzed using Nanoanalyser software (TA Instruments) to extract the thermodynamic parameters, enthalpy of binding (Δ*H*°, *K*_d_ (1/*K*_a_), and stoichiometry (*n*). *K*_d_, Δ*H*°, and *n* were obtained after fitting the integrated and normalized data to a single-site binding model. The apparent binding free energy (Δ*G*°) and entropy (Δ*S*°) were calculated from the relationships Δ*G*° = −*RT* ln(*K*_d_) and Δ*G*° = Δ*H*° − *T*Δ*S*°. All experiments were performed in triplicate to ensure reproducibility; standard deviations are reported from three runs (table S4).

### AlphaFold Multimer computational modeling and validation

To generate predicted models of the SNX27 and VARP complex, we used the AlphaFold2 Multimer neural network–based structural prediction method (*72*–*74*). For complex modeling, the sequence of human SNX27 FL (residues 1 to 541; UniProt database Q96L92) was modeled with mammalian VARP FL (residues 1 to 1050; UniProt database Q96NW4) or N-VARP alone (residues 1 to 117). AlphaFold version 2.3.2 (AF2.3.2) computations were executed using the resources of the Advanced Computer Center for Research and Education (ACCRE) at Vanderbilt. Structural alignments and images were generated with PyMOL (Schrodinger, US) or Chimera (*75*). In all AlphaFold2 Multimer predictions, we applied four criteria to evaluate model reliability (*72*–*74*): pLDDT scores for local structure accuracy, ipTM scores for the accuracy of the predicted relative positions of the subunits forming the protein-protein complex, PAE scores for the distance error between residues, and consistency among five top-ranked models for prediction convergence as judged by structural superposition. In most cases, consistency of top 5 aligned models agreed with the pLDDT, ipTM, and PAE criteria. The AlphaFold2 Multimer structural model was further validated using PISA (*67*) to evaluate the buried surface area and MolProbity (*68*) to evaluate protein geometry and clashes (table S2).

### Tissue culture and transfection

VARP-pEGFP-N1 was transiently overexpressed in the Expi293 Expression System (Thermo Fisher Scientific). Cells were grown in 125-ml flasks, divided to a final titer of 3 × 10^6^ cells/ml, and then transfected with 1.0 mg of VARP-pEGFP-N1 plasmid DNA per milliliter of culture using the ExpiFectamine 293 kit (Thermo Fisher Scientific). As a control, a similar amount of empty pEGFP-N1 vector was also transfected in the Expi293 Expression System. Cells were harvested at 48 hours posttransfection for use in immunoprecipitation assays.

### Immunoprecipitation assay

Cell lysates were obtained from untransfected, empty vector (pEGFP-N1)–transfected, and VARP-pEGFP-N1–transfected cells after resuspension and lysis in buffer [10 mM Hepes, pH 7.5, 150 mM NaCl, 0.5 mM EDTA, 1% NP-40, and 1 cOmplete Mini EDTA-free Protease Inhibitor Cocktail tablet (Roche)]. Cell slurry was vortexed briefly, passed 10 times through a 23G needle, incubated on ice for 30 min, and centrifuged at 20,500*g* for 30 min. The soluble lysate fraction was reserved, and the total protein concentration was measured using Precision Red (Cytoskeleton). Input samples were prepared from the reserved lysate. Lysate samples were incubated with unconjugated agarose (control) resin (Chromotek) for 30 min at 4°C to reduce background binding. Samples from untransfected, empty vector, and VARP-pEGFP-N1 precleared lysates were divided equally among GFP-trap resin (Chromotek) and fresh control resin for coimmunoprecipitation experiments. Dilution buffer (20 mM Hepes, pH 7.5, 150 mM NaCl, 0.5 mM EDTA, and 1 cOmplete Mini EDTA-free Protease Inhibitor Cocktail tablet per 20 ml) was added to bring each sample to an equal volume, and samples were incubated for 1 hour at 4°C followed by three washes in dilution buffer. Samples were eluted from resin by adding SDS loading buffer to the washed resin pellet and boiling for 5 min at 95°C. Samples were subjected to SDS-PAGE using 4 to 20% gels (Bio-Rad) and transferred to polyvinylidene difluoride membranes (Immobilon-P; Millipore). Blots were incubated with indicated primary and HRP-conjugated secondary antibodies and detected using SuperSignal West Pico PLUS Chemiluminescent Substrate (Thermo Fisher Scientific). All uncropped Western blot images from coimmunoprecipitation experiments are displayed in figs. S13 and S14.
