# Supplementary material for: VARP binds SNX27 to promote endosomal supercomplex formation on membranes
Source: Sci Adv. 2025 Feb 12;11(7):eadr9340. doi: 10.1126/sciadv.adr9340 (PMC11817943; doi:10.1126/sciadv.adr9340)
Supplement: Supplementary file 1 — Figs. S1 to S14 Tables S1 to S5 [file sciadv.adr9340_sm.pdf]

Science Advances
AAAS

Supplementary Materials for

VARP binds SNX27 to promote endosomal supercomplex formation on membranes

Mintu Chandra *et al.*

Corresponding author: Lauren P. Jackson, lauren.p.jackson@vanderbilt.edu

Sci. Adv. **11**, eadr9340 (2025)
DOI: 10.1126/sciadv.adr9340

This PDF file includes:

Figs. S1 to S14
Tables S1 to S5

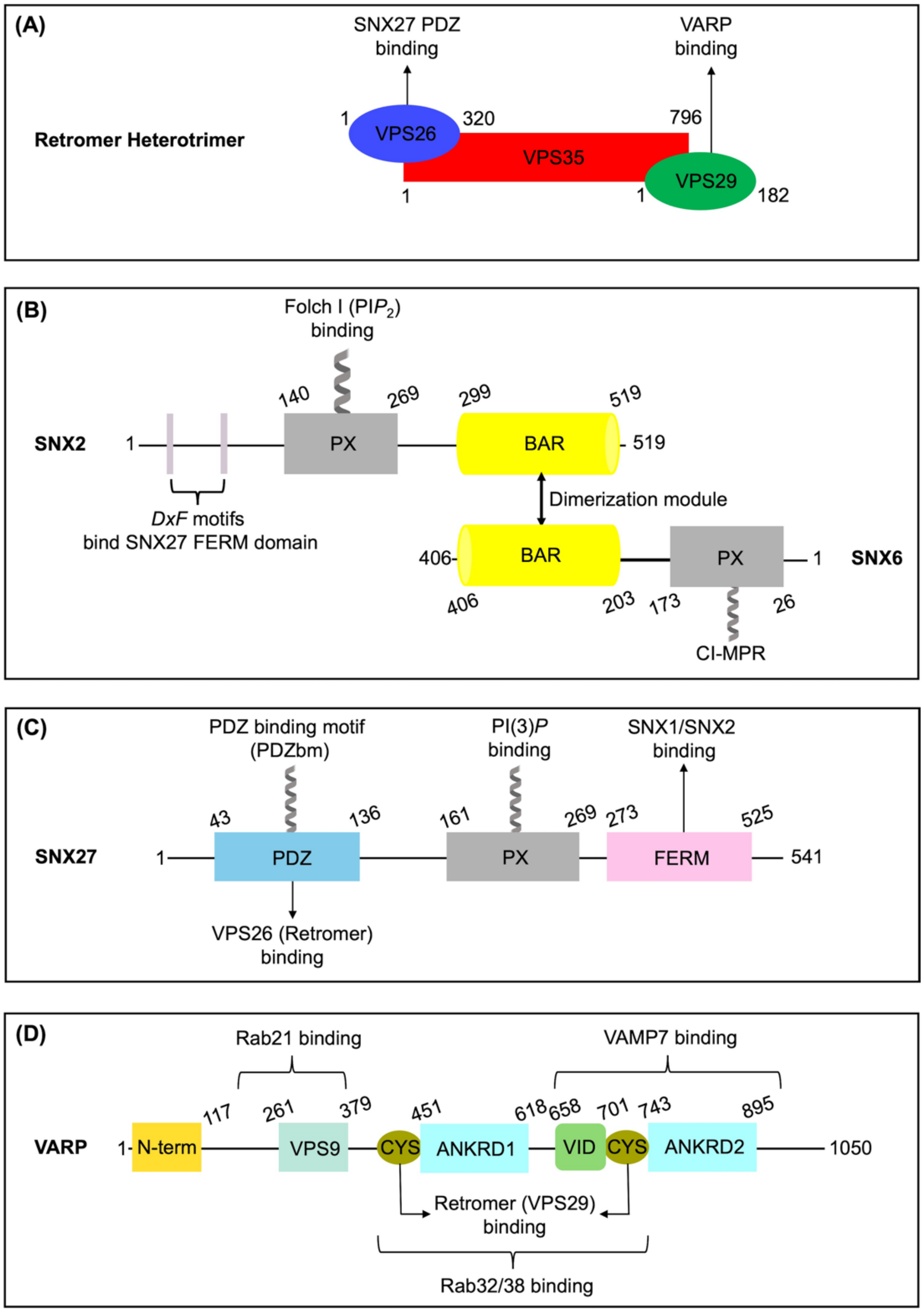

fig. S1. Domain architecture of endosomal proteins used in this study. **(A)** Domain architecture of mammalian Retromer containing three Vacuolar Protein Sorting (VPS) subunits: VPS26, VPS35, and VPS29. **(B)** Domain architecture of mammalian SNX2/SNX6 (ESCPE-1). The SNX2 N-terminus is an extended flexible region; the PX domain binds phospholipids; and the C-terminal BAR domain forms a dimer with SNX6. SNX6 contains a short and flexible N-terminus and a PX domain known to recognize a motif in CI-MPR cargo; and a C-terminal BAR domain. **(C)** Human SNX27 contains an N-terminal PDZ; central PX; and C-terminal FERM domains. The PDZ domain binds transmembrane proteins containing a PDZ binding motif (PDZbm) and the VPS26 subunit of Retromer. The PX domain recognizes PI(3)*P*, a the FERM module has been shown to bind flexible N-terminal regions of two SNX-BAR proteins, SNX1/SNX2. **(D)** Human VARP (also known as ANKRD27) domains are shown with established binding partners highlighted. Two cysteine-rich motifs (CYS) engage the VPS29 Retromer subunit, and this study establishes how the VARP N-terminus binds SNX27.

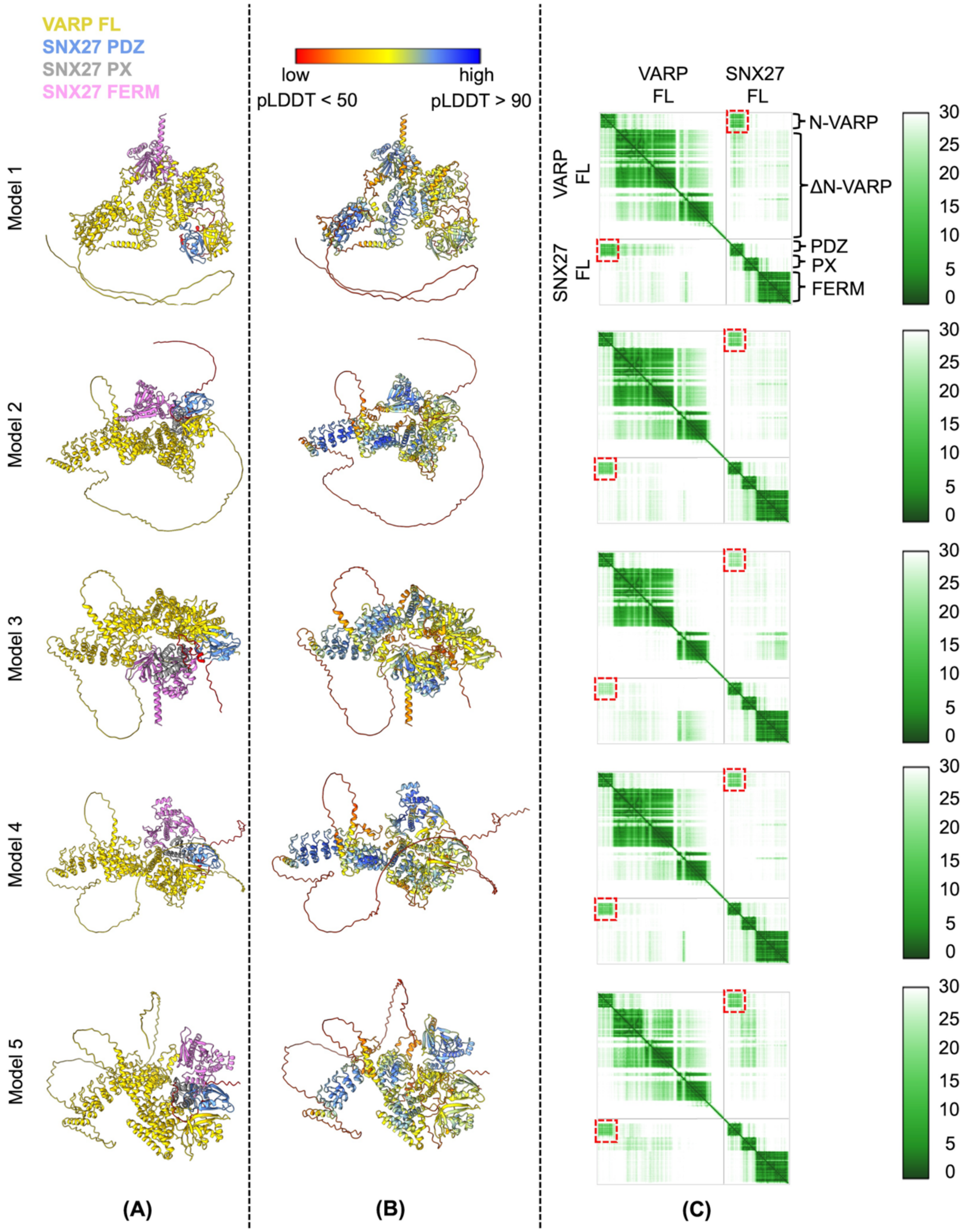

fig. S2. AlphaFold models of full-length VARP and full-length SNX27. (A) Ribbon diagrams of the five top-ranked models generated in AlphaFold2.3 Multimer depicting full-length VARP bound to full-length SNX27. VARP is shown in gold ribbons with SNX27 colored by domain: SNX27 PDZ in sky blue; SNX27 PX in grey, and SNX27 FERM in magenta color. **(B)** Ribbon diagrams of the top five models colored by pLDDT score. High pLDDT scores (shown in blue) reflect high confidence in local structure prediction. **(C)** For each model, the Predicted Aligned Error (PAE) score matrix is shown. Low scores (dark green color) represent high confidence in the relative position in 3D space (right column). The boundaries of protein domains can be observed in the PAE plots, including N-VARP (residues 1-117); ΔN-VARP (residues 118-1050); SNX27 PDZ (residues 43-136); SNX27 PX (residues 161-269); SNX27 FERM (residues 273-525). The predicted interaction between N-VARP and the SNX27 PDZ domain is highlighted as red dashed boxes on the PAE plots.

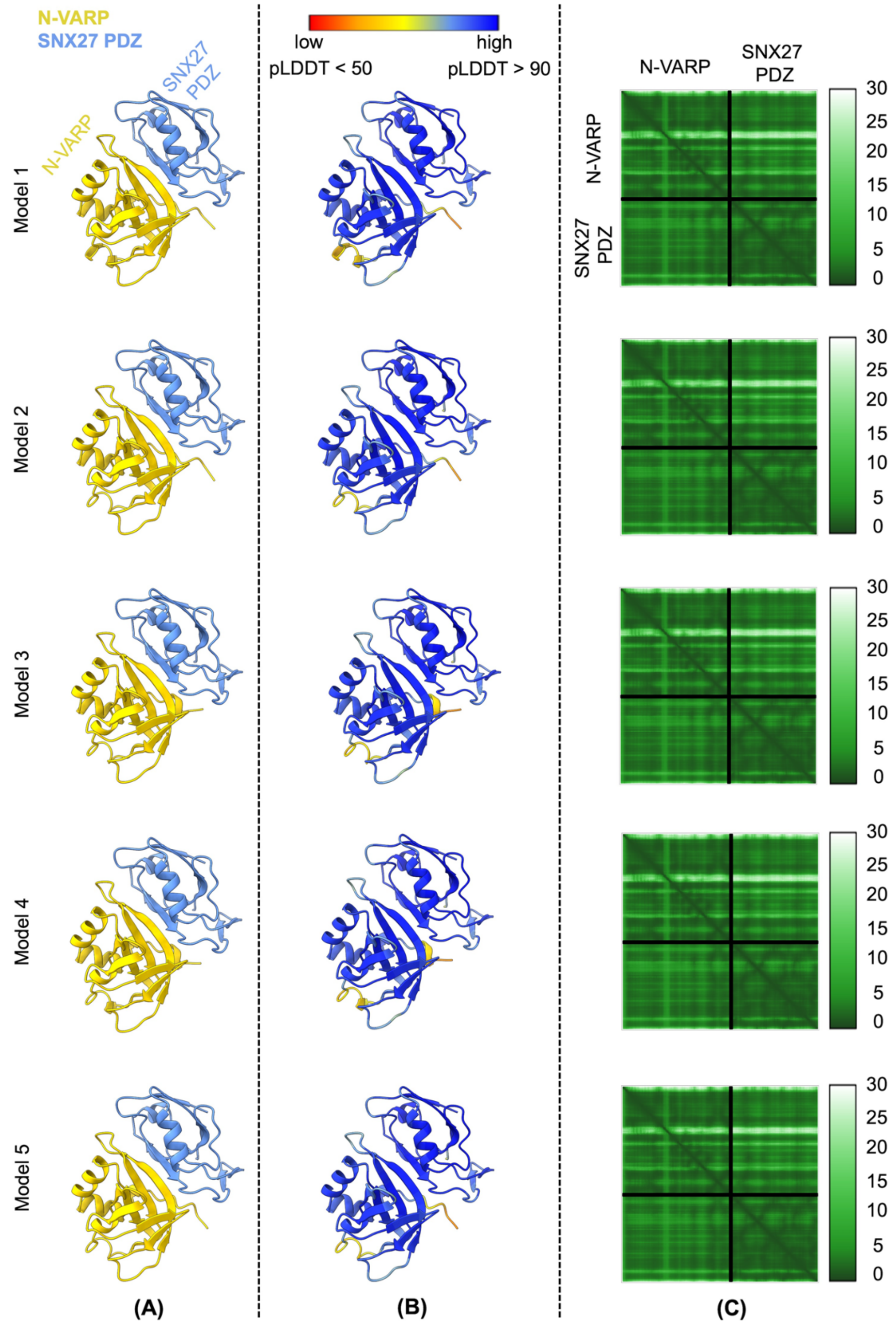

fig. S3. AlphaFold models of the VARP N-terminal globular domain with SNX27 PDZ domain. (A) Ribbon diagrams of the five top-ranked AlphaFold2.3 Multimer models depicting N-VARP bound to SNX27 PDZ. Models are colored by domain, with N-VARP in gold and SNX27 PDZ in sky blue. **(B)** Ribbon diagrams of top five models colored by pLDDT score; dark blue (scores >90) represents high confidence in local prediction. **(C)** For each model, the Predicted Aligned Error (PAE) score matrix is shown. The PAE score matrix provides low scores (deep green), signifying high confidence in the relative position in 3D space The boundaries of protein domains (N-VARP and SNX27 PDZ) are labeled in the PAE plot. Overall, AlphaFold consistently generates the same predicted model for this interaction.

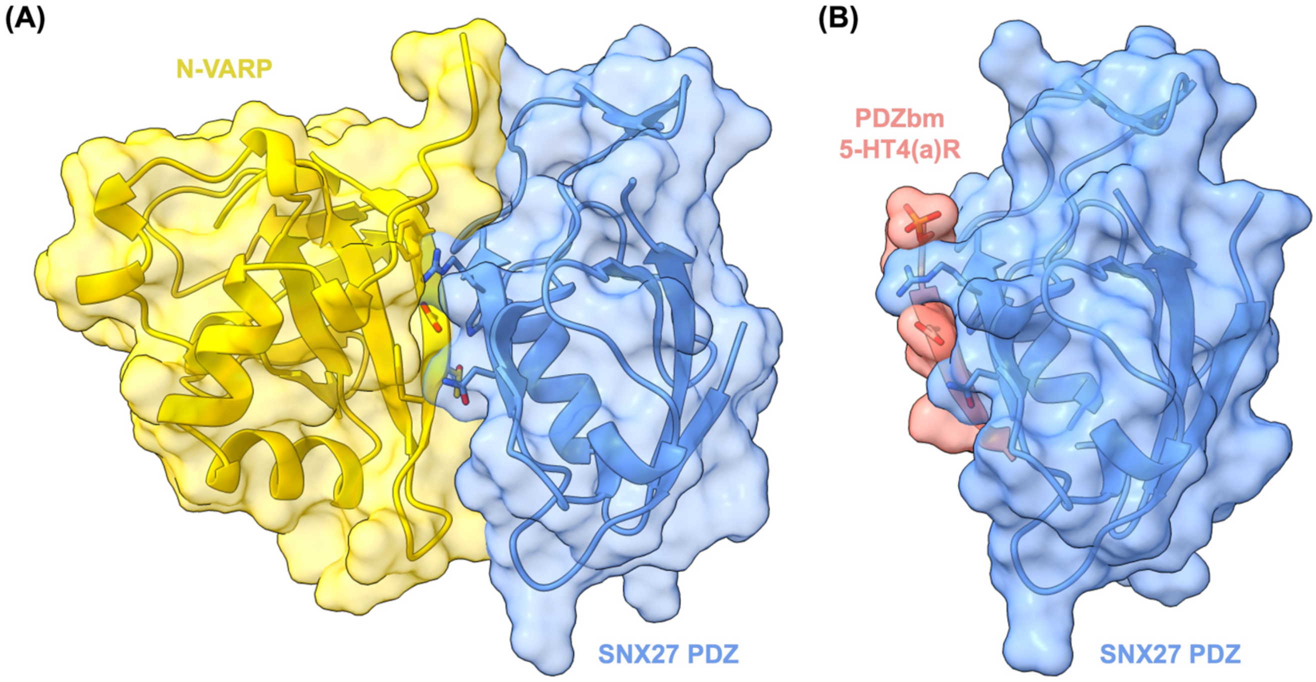

(C) Buried Surface Area comparison:

Protein-complex	Buried Surface Area (Å^2)<br>(calculated by PISA server)
PDB ID 5EM9<br>5-HT4(a)R:SNX27 PDZ	1030.0
AlphaFold2.3 Multimer<br>N-VARP:SNX27 PDZ	1506.4

fig. S4. Comparative analysis of interactions between SNX27 PDZ and the VARP N-terminus or PDZ binding motif (PDZbm) cargo peptide. (A) Transparent surface view is shown over a ribbon diagram of VARP N-terminus (gold) and SNX27 PDZ domain (sky blue) model from AlphaFold. **(B)** Equivalent transparent surface view is shown over a ribbon diagram of the experimental X-ray structure with PDZbm peptide from 5-HT4(a)R (light red) bound to SNX27 PDZ domain (sky blue). **(C)** Comparison of the predicted buried surface area between these two structures calculated in PISA. The interaction between VARP N-terminus and SNX27 PDZ buries 50% greater surface area than does the PDZbm cargo peptide, in agreement with observed dissociation constants.

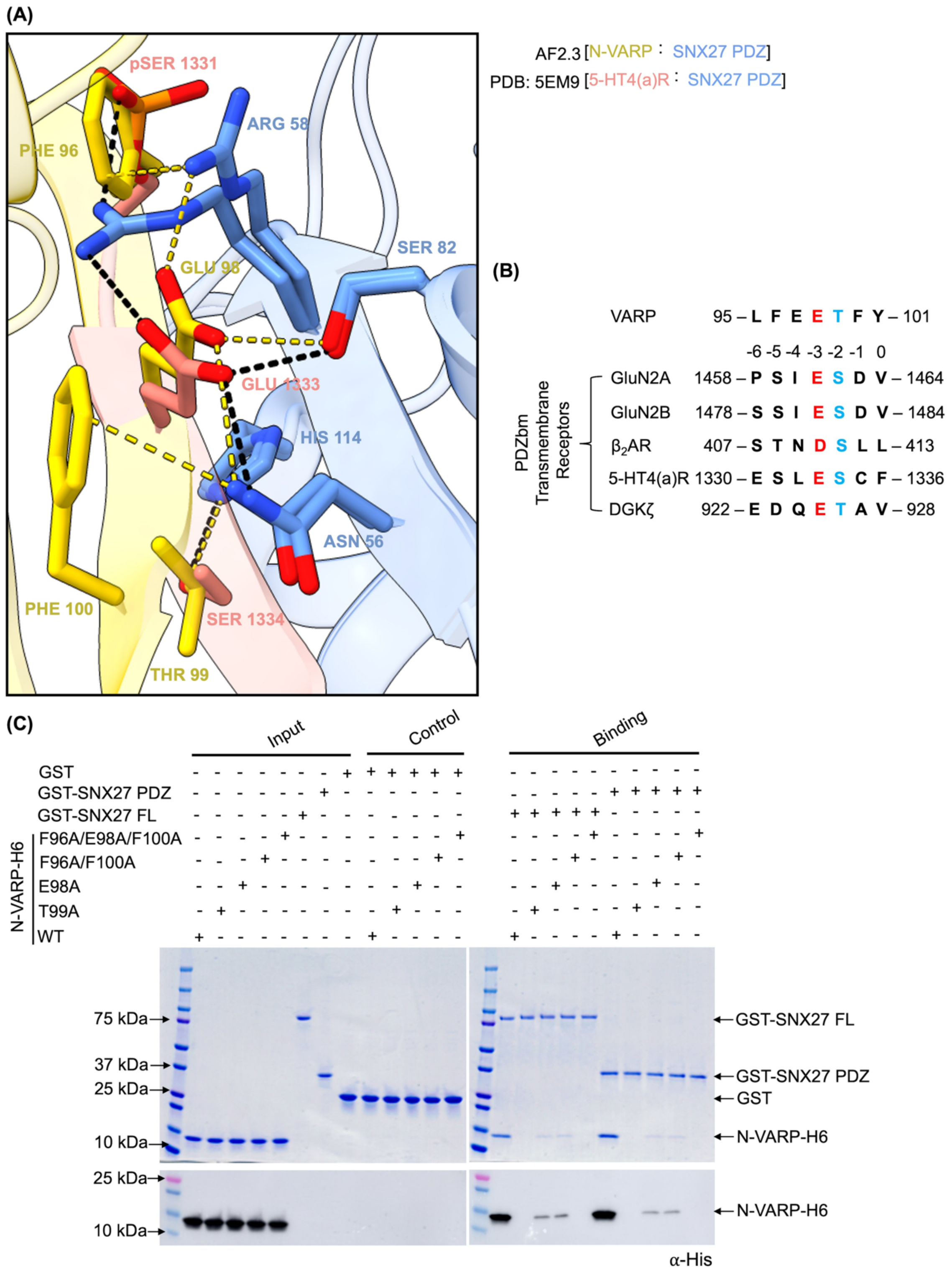

fig. S5. The VARP N-terminus and PDZbm cargoes bind in the same pocket on the SNX27 PDZ domain. **(A)** Close-up view of structural superposition between C-terminal PDZbm cargo from 5-HT4(a)R (light red side chains) and SNX27 PDZ domain (sky blue; PDB: 5EM9) and the AlphaFold-predicted model of N-terminal VARP (gold) bound to SNX27 PDZ (sky blue). The superposition reveals multiple overlapping residues that mediate both interactions. Both N-terminal VARP (residues Phe96, Glu98, Thr99 and Phe100) and phosphorylated PDZbm motif (residues phospho-Ser1331, Gle1333 and Ser1334) interact with the same patch

on the SNX27 PDZ domain composed of residues Asn56, Arg58, Ser82, and His114. Predicted interaction distances between the VARP N-terminus and SNX27 PDZ domain are represented as yellow dashed lines, while distances determined from the experimental structure of the PDZbm cargo motif and SNX27 PDZ domain (PDB ID: 5EM9) are shown as black dashed lines. **(B)** Sequence alignment and comparison of VARP N-terminus (motif: LFEETFY; residues 95−101) and multiple PDZ binding motifs in five known transmembrane receptors. The motif position numbers are assigned according to the classical type I PDZbm sequence ($D/E^{-3}-S/T^{-2}-X^{-1}-\Phi^{0}$, where Φ represents any hydrophobic residue). Residues corresponding to -2 and -3 positions are highlighted in blue and red, respectively. **(C)** GST pulldown experiments confirm VARP N-terminal residues from AlphaFold2 model are involved in binding. GST-tagged full length SNX27 or GST-SNX27 PDZ domain were used as baits with purified His-tagged N-terminal VARP mutant proteins (E98A; T99A; F96A/F100A double mutant; and F96A/E98A/F100A triple mutant) as prey. Representative SDS-PAGE gel stained with Coomassie blue is shown in top panel with α-His Western blot shown in bottom panel.

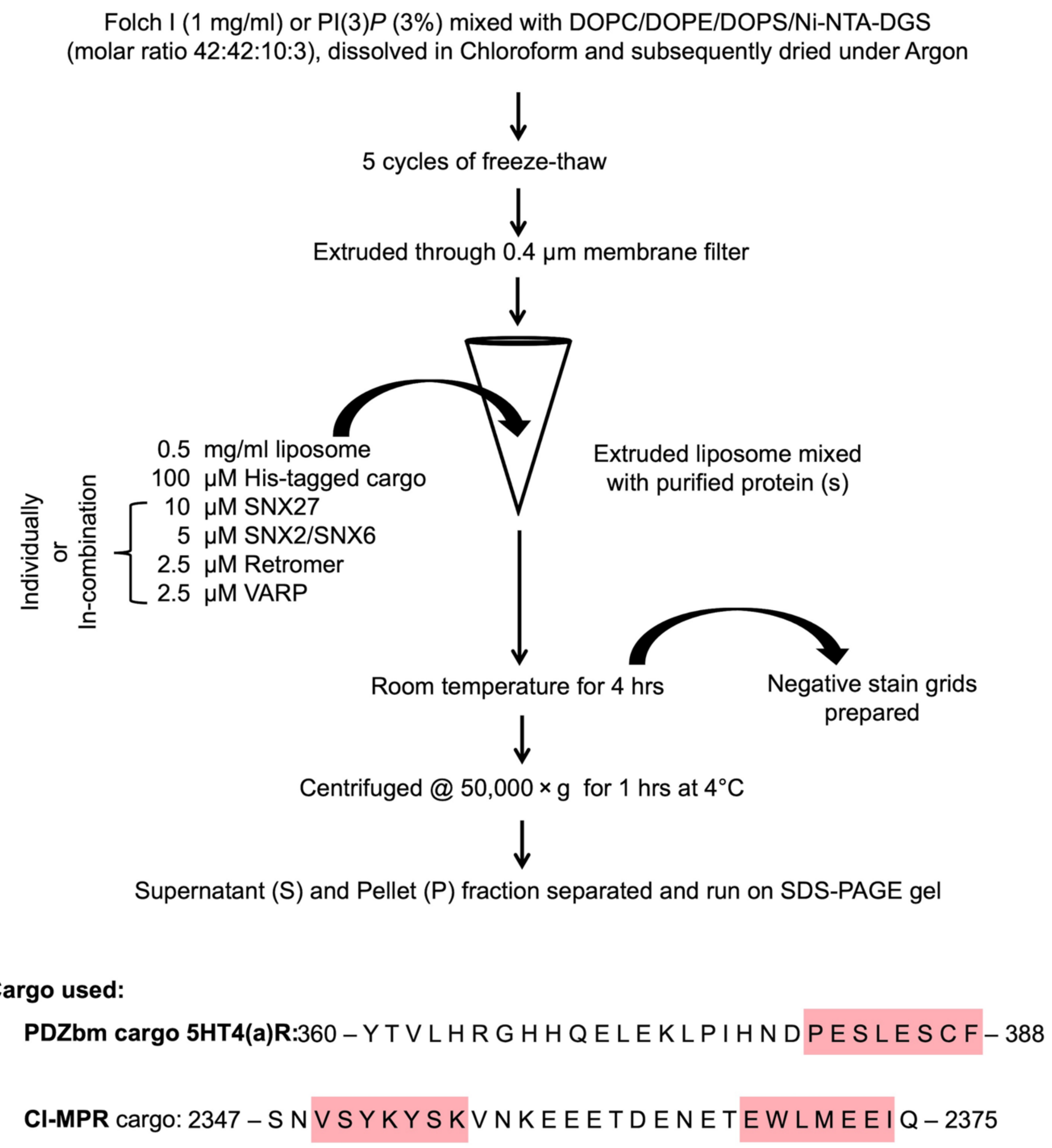

fig. S6. Flow chart depicting steps in liposome preparation and liposome pelleting assay.

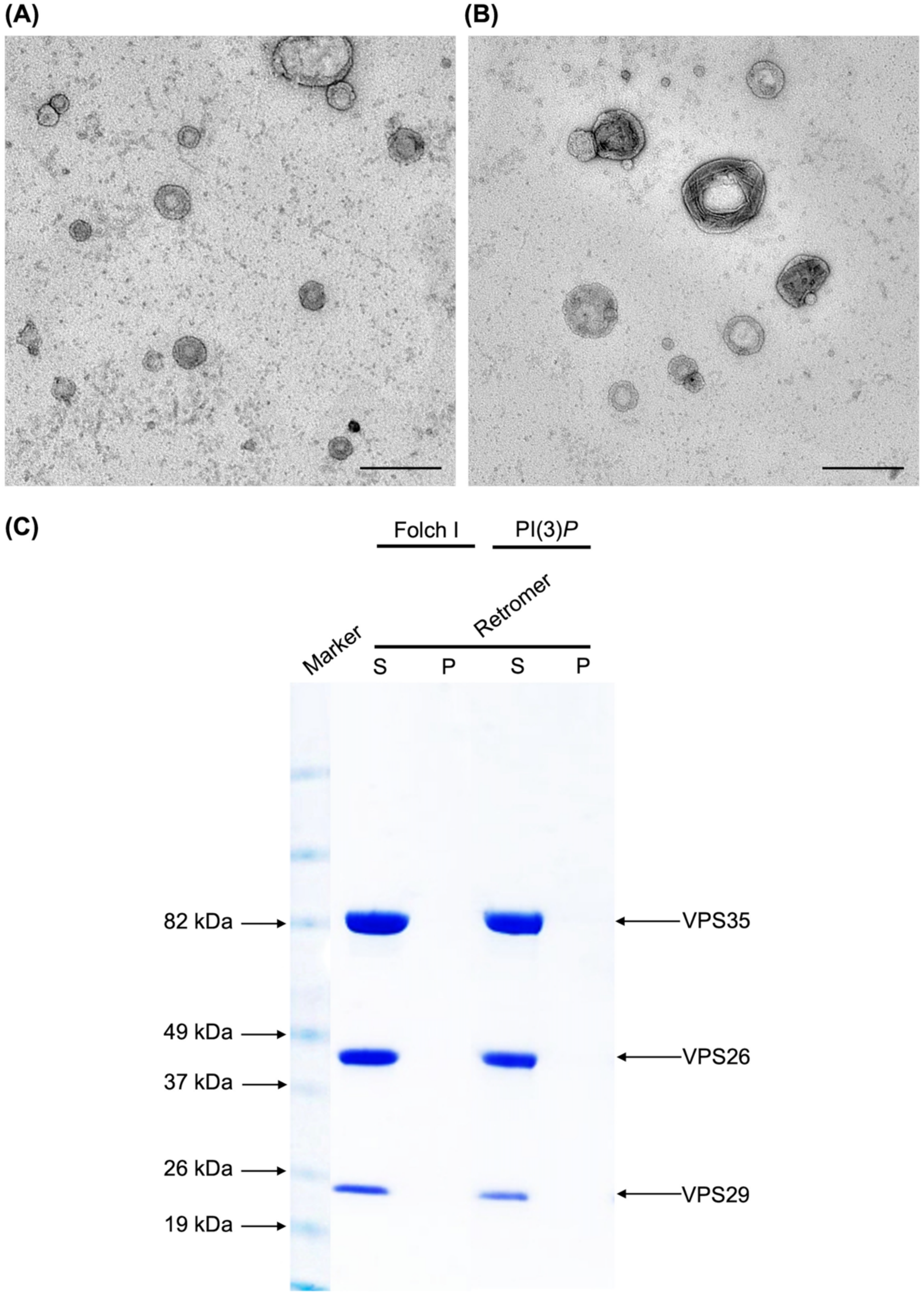

fig. S7. Control experiments to establish the reconstitution system. Representative negative stain EM images of liposomes containing **(A)** PI(3)*P* and **(B)** Folch I as controls. Liposomes containing these phospholipid compositions do not exhibit tubules. Liposomes containing either **(A)** PI(3)*P* or **(B)** Folch I were incubated with buffer (20 mM HEPES-KOH pH 7.5, 200 mM NaCl, and 1 mM Tris (2-carboxyethyl)phosphine) and visualized using negative stain EM. (Scale bar = 500 nm). **(C)** Liposome pelleting assay of purified Retromer complex on liposomes enriched with Folch I or PI(3)*P*. Samples were subjected to ultracentrifugation followed by SDS-PAGE and Coomassie staining of the unbound supernatant (S) and bound pellet (P) fractions. Retromer is not recruited to membranes in the absence of cargo or SNX27.

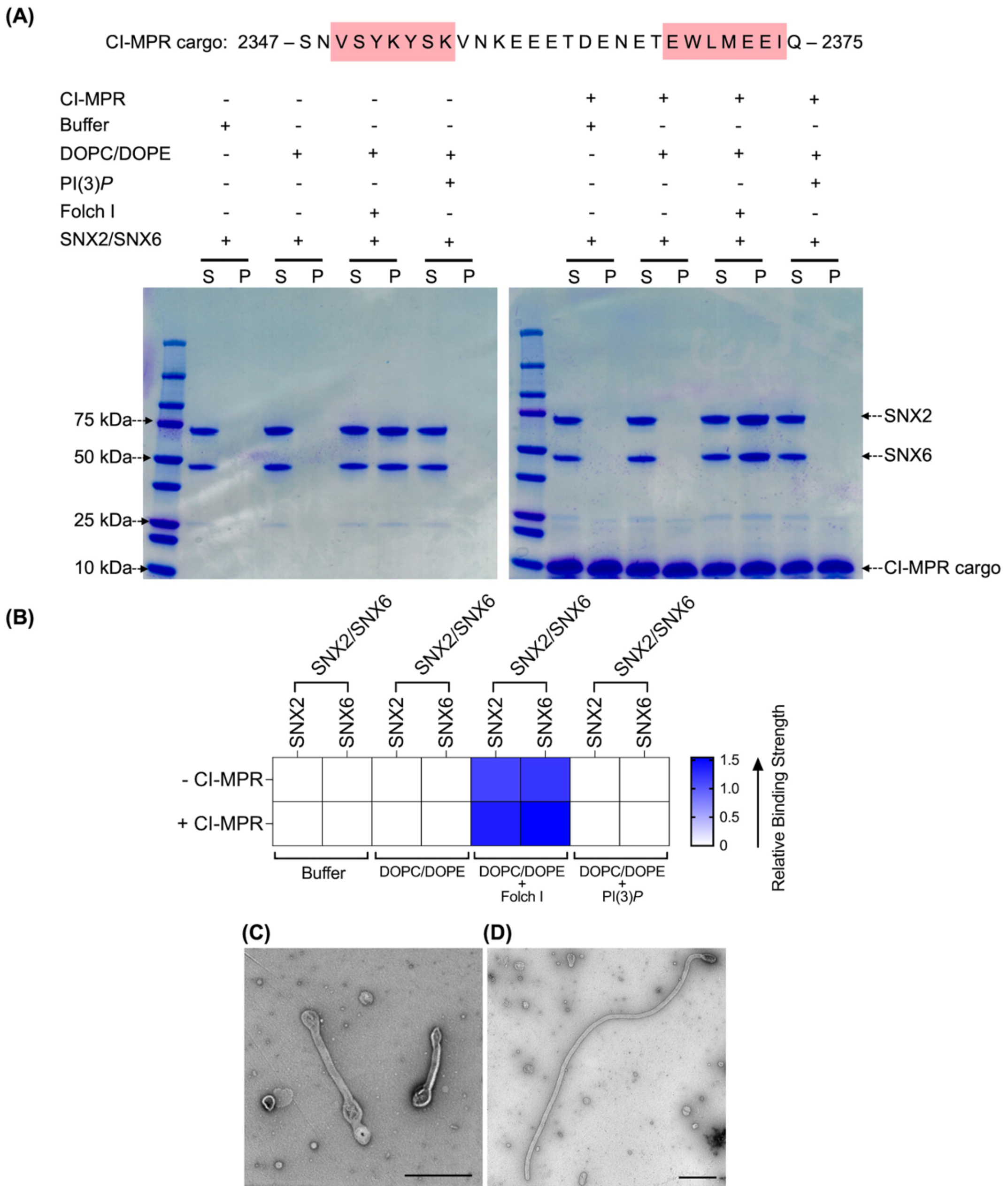

fig. S8. Membrane binding and tubulation properties of SNX2/SNX6 differ from those of SNX27/ Retromer. (A) Membrane binding of human SNX2/SNX6 (ESCPE-1) complex by liposome pelleting assay. Purified human SNX2/SNX6 complex was incubated with liposomes in the presence or absence of the CI-MPR cargo motif (residues 2347–2375; sequence motifs highlighted in red text). Buffer and DOPC/DOPE alone were used as negative controls to detect non-specific binding. Samples were subjected to ultracentrifugation followed by SDS-PAGE and Coomassie staining of the unbound supernatant (S) and bound pellet (P) fractions. SNX2/SNX6 is recruited to membranes in the presence of Folch I alone (left gel) and Folch I with CI-MPR cargo (right gel). **(B)** Binding of SNX2/SNX6 to phosphoinositide-enriched membranes visualized by SDS-PAGE was quantified by measuring relative protein band intensities (ImageJ) as in Fig. 3. **(C, D)** Negative stain EM reveals tubulation of Folch I-enriched liposomes incubated with SNX2/SNX6 (ESCPE-1) alone **(C)** or in presence of CI-MPR cargo **(D).** Scale bars represent 500 nm.

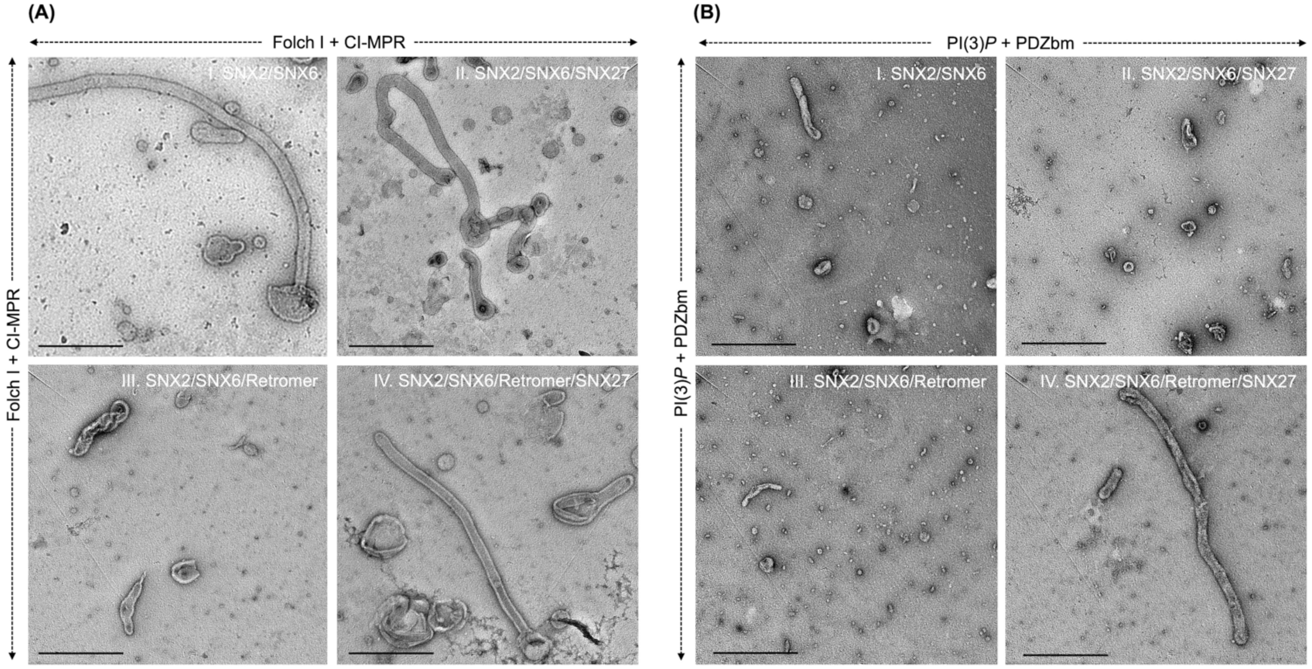

fig. S9. Morphology of membrane tubules generated by endosomal coat proteins visualized using negative stain EM. (A) Representative negative stain EM images depicting Folch I-enriched liposomes with CI-MPR cargo motif following incubation with (I) SNX2/SNX6; (II) SNX2/SNX6 and SNX27; (III) SNX2/SNX6 and Retromer; and (IV) SNX2/SNX6, Retromer, and SNX27. These data further suggest SNX2/SNX6 drives tubulation on its own without contribution from SNX27 or Retromer. SNX27 does not effectively bind Folch I-enriched membranes these conditions (see Fig. 4A) or contribute to morphology. Retromer does not pellet with SNX2/SNX6 under these conditions (Fig. 4A), and its presence may negatively impact tubule formation (panel III). **(B)** Representative negative stain EM images depicting PI(3)*P*-enriched liposomes with 5-HT4(a)R PDZbm cargo motif following incubation with (I) SNX2/SNX6; (II) SNX2/SNX6 and SNX27; (III) SNX2/SNX6 and Retromer; and (IV) SNX2/SNX6, Retromer, and SNX27. SNX2/SNX6 is not efficiently recruited to PI(3)*P*-enriched membranes (see Fig. 4B) and probably does not contribute to morphology observed in panel B-IV. Scale bars represent 500 nm.

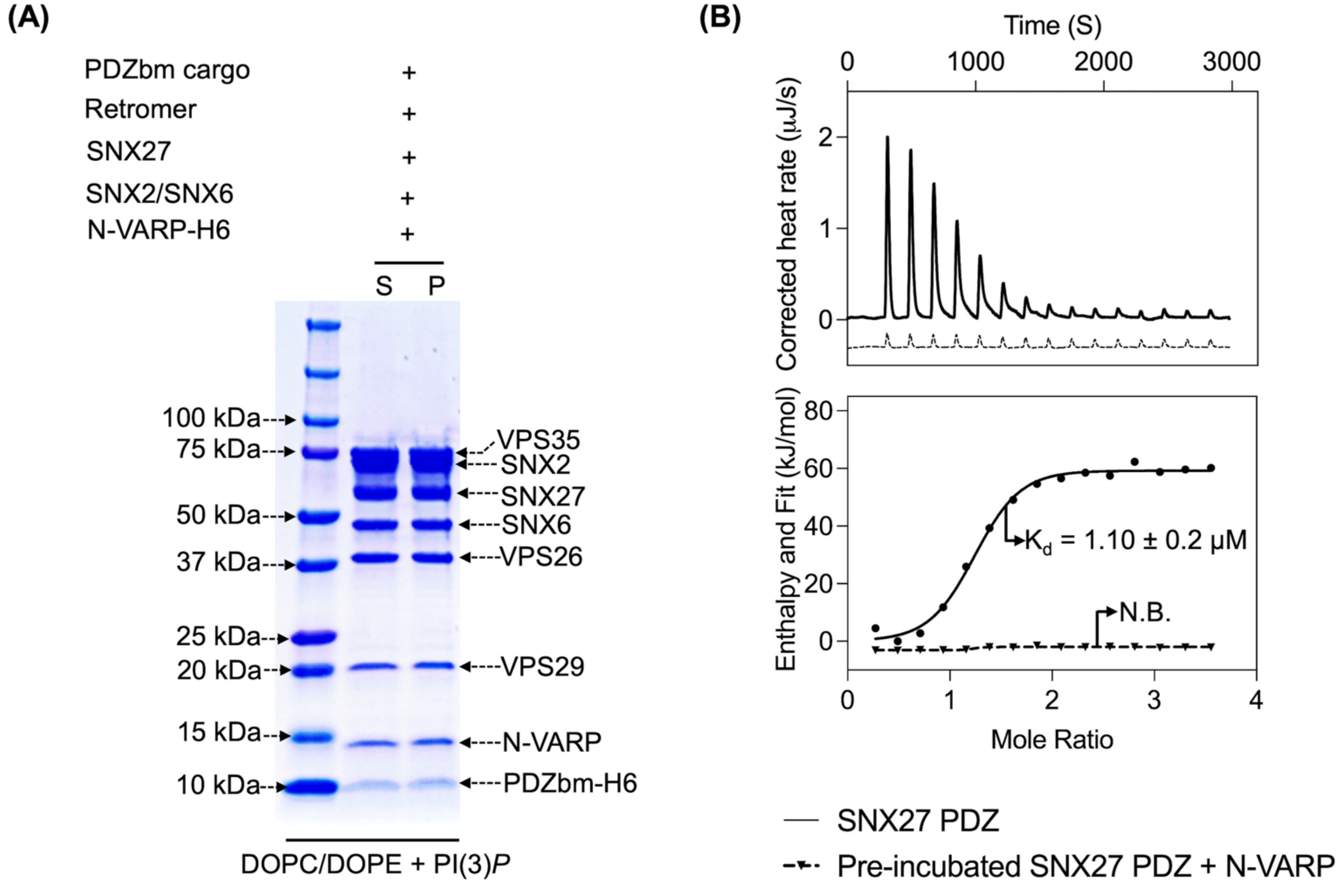

fig. S10. The VARP N-terminus recruits the supercomplex to membranes and binds in SNX27 PDZ cargo binding site. (A) Purified N-VARP protein was incubated with SNX27, SNX2/SNX6, and Retromer in the presence of PDZbm cargo and PI(3)*P*-enriched liposomes. All protein components are recruited to membranes in the presence of N-VARP alone. **(B)** Isothermal titration calorimetry (ITC) competition experiments were undertaken to establish whether N-VARP and PDZbm cargo motifs bind the same site on SNX27 PDZ. Synthesized PDZbm cargo peptide from 5-HT4(a)R was titrated into the calorimeter cell containing either purified SNX27 PDZ protein alone (dark black traces) or a 1:1 mixture of purified SNX27 PDZ and N-VARP proteins (dotted black traces). The PDZbm motif binds SNX27 PDZ with a K_D near 1 μM as established in the literature, while no detectable binding is observed when PDZbm peptide is titrated into the SNX27 PDZ/N-VARP mixture.

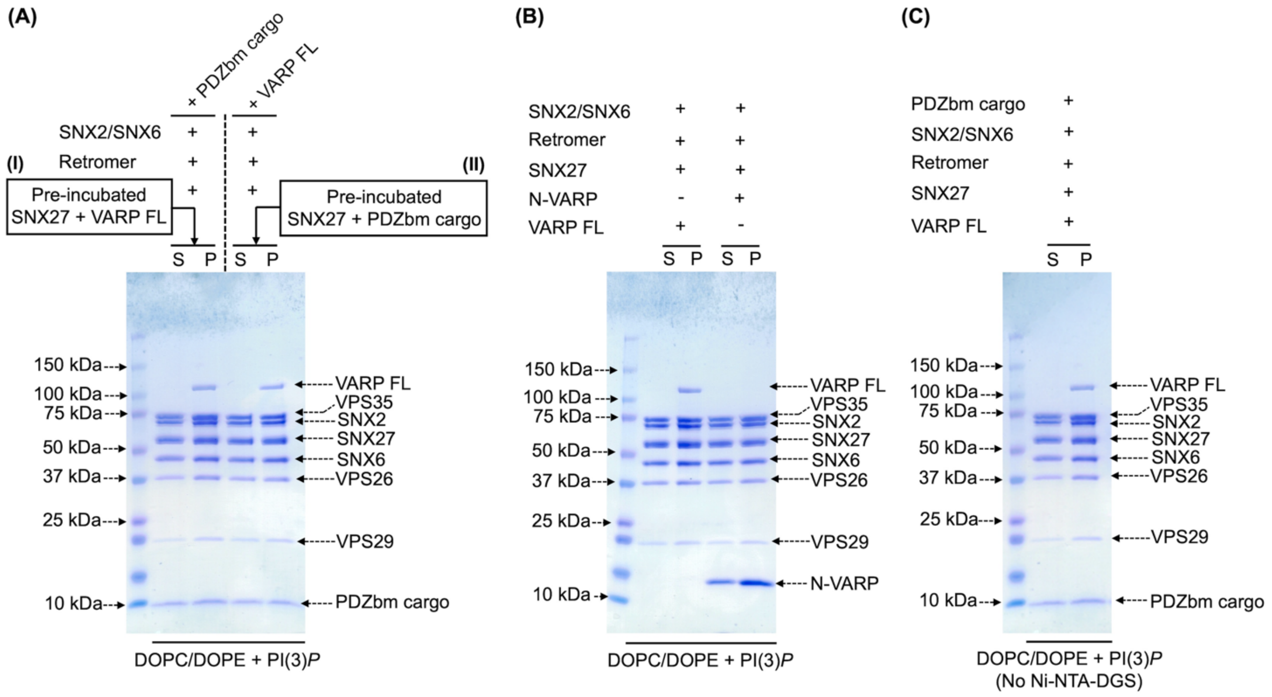

fig. S11. Competition and comparative experiments to address PDZbm cargo incorporation into assembled endosomal coats. (A) Liposome pelleting competition experiments, in which SNX27 is pre-incubated either with full-length VARP (FL) (I) or with cargo (II), demonstrate all components of the endosomal supercomplex efficiently pelleted in the presence of PDZbm cargo. In both versions, sub-stoichiometric amounts of full-length VARP were observed in pellet fractions. This contrasts with experiments using N-VARP (Fig. 6B – I and II), in which N-VARP either pelleted in stoichiometric amounts (Fig. 6B-II) or in excess (Fig. 6B-I). **(B)** Liposome reconstitution experiments with either full-length VARP (VARP FL) or N-VARP conducted in the absence of cargo. These data reveal VARP binds sub-stoichiometrically in the complex, while N-VARP binds approximately stoichiometrically. Compared to experiments with cargo (Fig. 5A), there is no substantial difference in full-length VARP binding to membranes lacking cargo. In contrast, N-VARP displayed increased binding to PI(3)*P* liposomes when cargo was absent. This supports the hypothesis that full-length VARP binds sub-stoichiometrically, likely because one full-length VARP interacts with two Retromer VPS29 subunits, while each N-VARP can bind one SNX27 PDZ in the absence of PDZbm cargo. **(C)** Reconstitution experiments using PI(3)*P* liposomes without Ni-NTA-DGS shows comparable levels of PDZbm cargo in pellet fractions, strongly suggesting PDZbm cargo is incorporated in assembled coats through binding to the SNX27 PDZ domain.

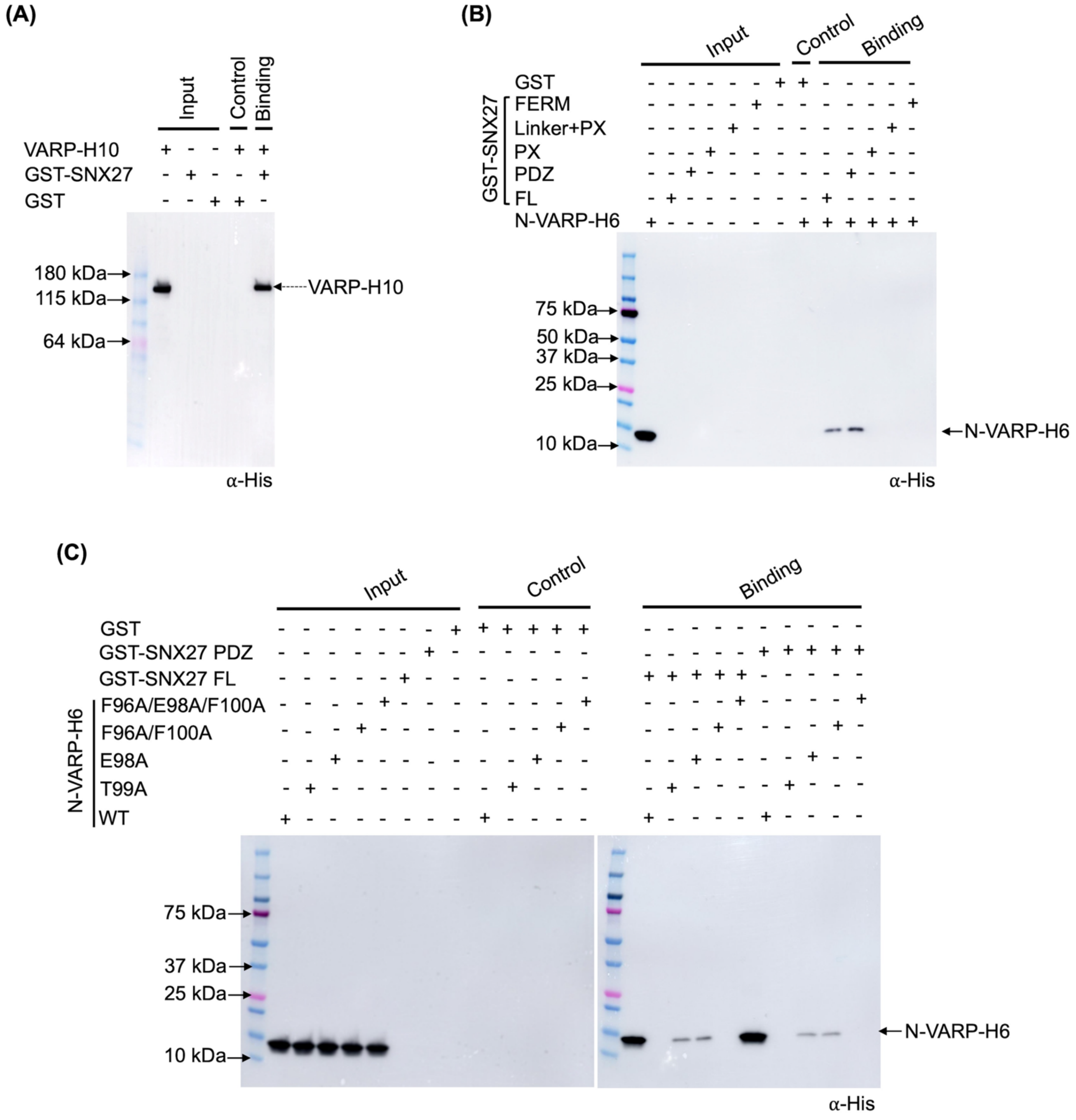

fig. S12. Uncropped Western blots associated with pulldowns presented in Fig. 1A, 2B; fig. S5C. Western blotting showing GST pulldown experiments with **(A)** purified GST-SNX27 as bait and His-tagged VARP-H10 as prey; **(B)** SNX27 full-length (FL), PDZ, PX, or FERM domains as bait and His-tagged N-VARP-H6 as prey; **(C)** GST-SNX27 FL or GST-SNX27 PDZ as bait and purified His-tagged N-VARP-H6 mutant proteins (E98A; T99A; F96A/F100A double mutant; and F96A/E98A/F100A triple mutant) as prey. Each of the blots were probed using α-His antibody (Abcam, ab184607).

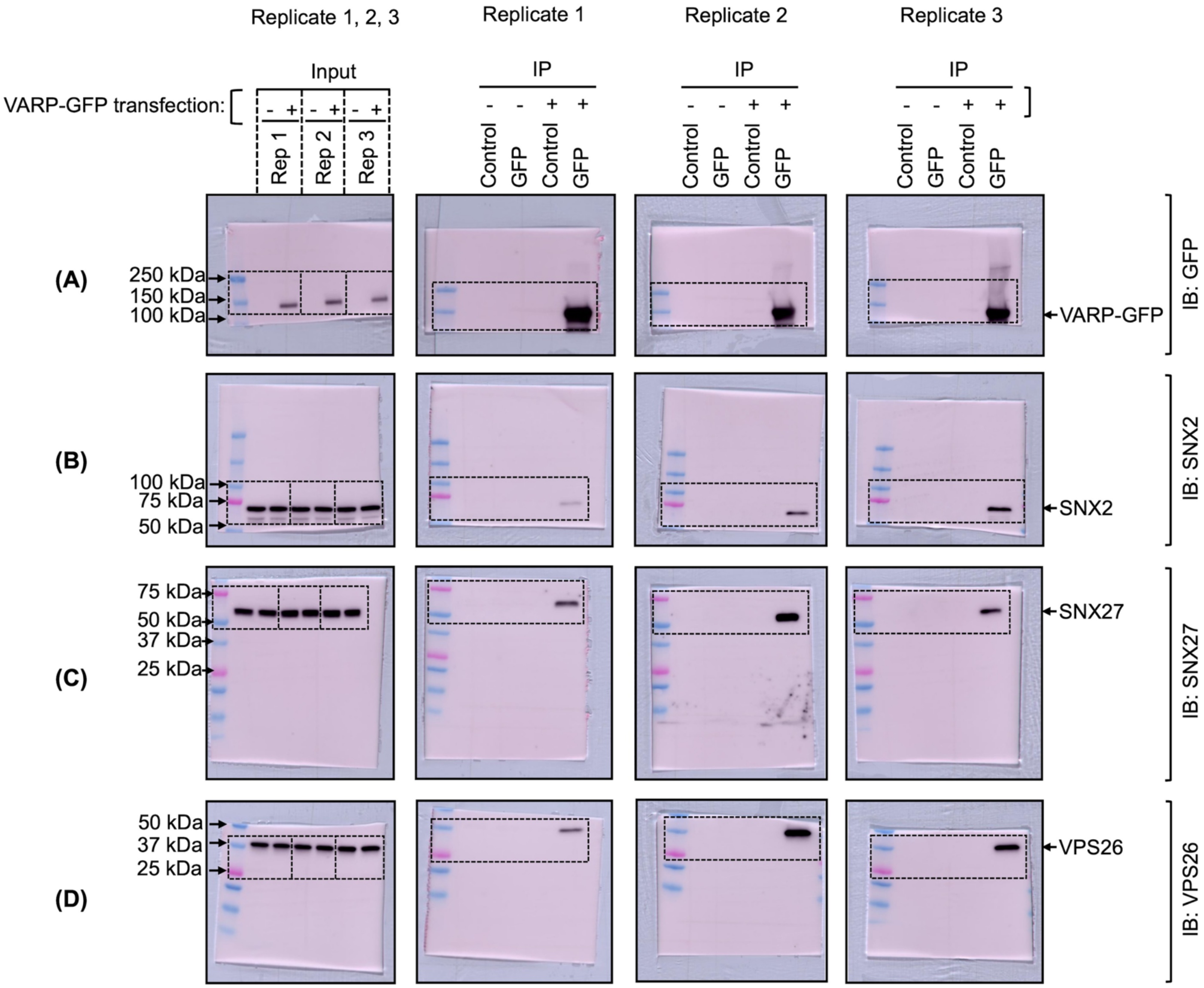

fig. S13. Uncropped Western blots from VARP-GFP transfected cell lysates associated with Fig. 5C. Co-immunoprecipitation experiments using full-length VARP-GFP overexpressed in Expi293 cells. The cell lysates were subjected to GFP trap–based immunoprecipitation and blotted against **(A)** GFP (ab6663; Abcam); **(B)** SNX2 (ESCPE-1 component; clone 13; 5345661; BD Biosciences); **(C)** SNX27 (ab77799; Abcam); and **(D)** VPS26 (Retromer subunit; 23892; Abcam). Each IP was performed in triplicate. Dashed boxes denote regions cropped for the indicated main figure.

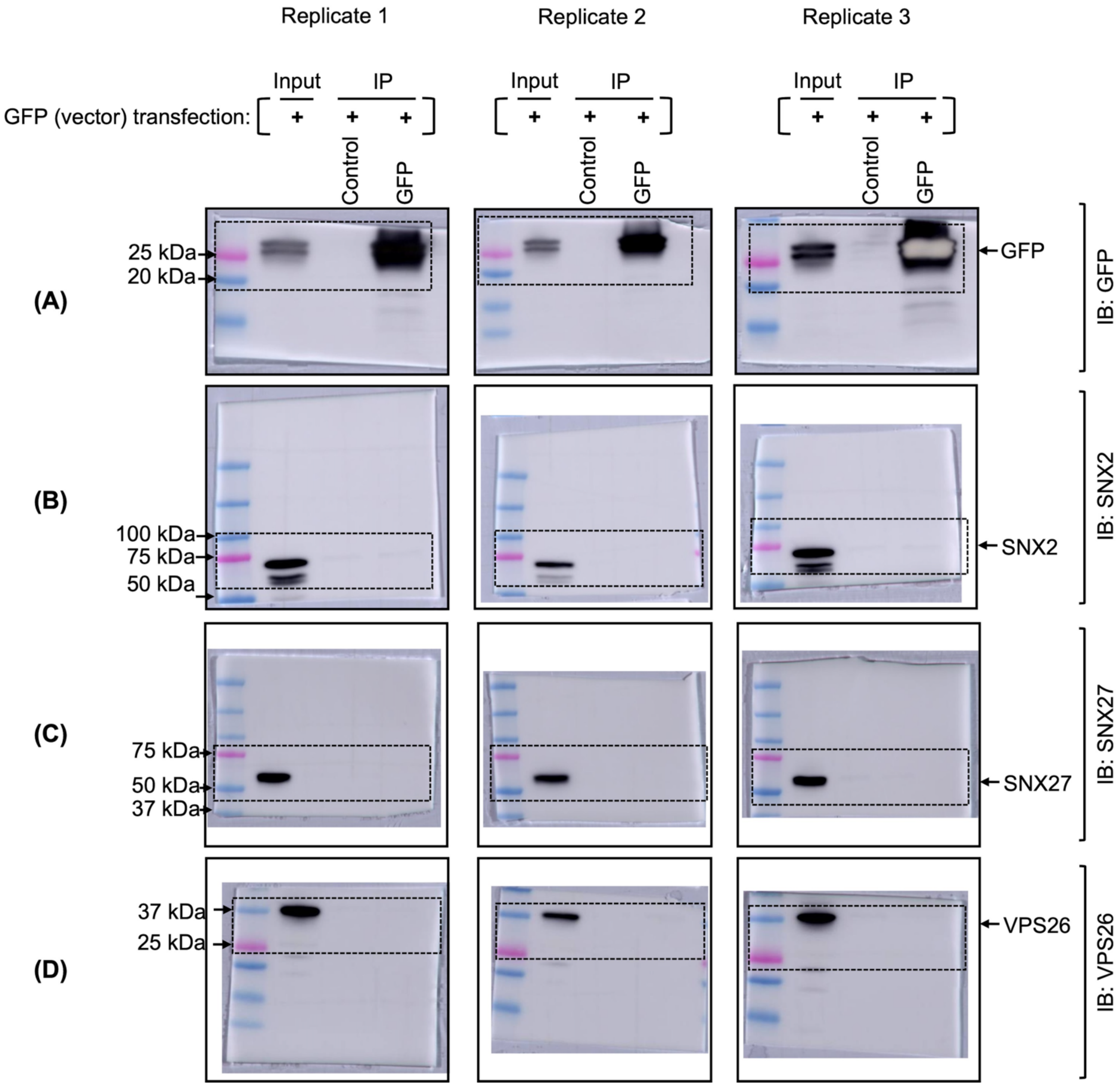

fig. S14. Uncropped Western blots from GFP transfected cell lysates associated with Fig. 5C. Co-immunoprecipitation experiments using empty pEGFP-N1 vector overexpressed in Expi293 cells. The cell lysates were subjected to GFP trap–based immunoprecipitation and blotted against **(A)** GFP (ab6663; Abcam); **(B)** SNX2 (ESCPE-1 component; clone 13; 5345661; BD Biosciences); **(C)** SNX27 (ab77799; Abcam); and **(D)** VPS26 (Retromer subunit; 23892; Abcam). Each IP was performed in triplicate. Dashed boxes denote regions cropped for the indicated main figure.

Loading Protein	Loading Protein Conc. (mg/ml)	Sample Protein	Sample Protein Conc. (μM)	K_D (μM)	k_{a1} (1/μMs)	k_{a2} (1/μMs)	k_{dis1} (1/s)	k_{dis2} (1/s)	RSS	R^2	Binding Model
VARP FL	0.05	Retromer	0.25 – 2	0.07 ± 0.01	$5.04*10^{-3} \pm 1.88*10^{-5}$	$5.35*10^{-8} \pm 7.63*10^{-9}$	$3.63*10^{-4} \pm 5.77*10^{-6}$	$8.91*10^{-3} \pm 9.33*10^{-4}$	3.33	0.99	1:2
VARP FL	0.05	SNX27 FL	0.50 – 4	0.34 ± 0.03	$2.24*10^{-3} \pm 9.91*10^{-6}$	N.A.	$7.54*10^{-4} \pm 4.73*10^{-6}$	N.A.	16.87	0.99	1:1
N-VARP	0.05	SNX27 FL	0.25 – 2	0.17 ± 0.02	$3.21*10^{-3} \pm 2.38*10^{1}$	N.A.	$5.68*10^{-4} \pm 7.99*10^{-6}$	N.A.	2.75	0.95	1:1
N-VARP	0.05	SNX27 PDZ	0.25 – 2	0.14 ± 0.03	$4.31*10^{-3} \pm 4.15*10^{1}$	N.A.	$6.42*10^{-4} \pm 1.35*10^{-5}$	N.A.	0.95	0.87	1:1
N-VARP	0.05	SNX27 PX	0.25 – 2	N.B.	N.B.	N.A.	N.B.	N.A.	N.B.	N.B.	N.B.
N-VARP	0.05	SNX27 FERM	0.25 – 2	N.B.	N.B.	N.A.	N.B.	N.A.	N.B.	N.B.	N.B.

table S1: Binding Kinetics calculated using the Octet R8 analysis software package

		PDB ID **5EM9** **5-HT4(a)R:SNX27 PDZ**	AlphaFold2.3 Multimer **N-VARP:SNX27 PDZ**
All atom contacts	Clashscore (all atoms)	5.03	2.44
Protein geometry	Poor rotamers	0 (0%)	2 (1.11%)
	Favored rotamers	82 (97.62%)	176 (97.78%)
	Ramachandran outliers	0 (0%)	0 (0%)
	Ramachandran favored	94 (96.91%)	201 (97.10%)
	Ramachandran distribution (Z-score)	0.64 ± 0.85	-0.12 ± 0.52
	MolProbity score[a]	1.45	1.22
	Cβ deviation >0.25 Å	0 (0%)	0 (0%)
	Bad bonds	0/792 (0%)	0/1655 (0%)
	Bad angles	0/1074 (0%)	4/2239 (0.18%)
Peptide omegas	Cis prolines	0/3 (0%)	0/9 (0%)
Additional validations	Chiral volume outliers	0/125	0/259

In the column results, the raw count is listed first with percentage in parentheses.

[a] MolProbity score combines the clashscore, rotamer, and Ramachandran evaluations into a single score.

table S2: Molprobity score comparison between the crystal structure of 5-HT4(a)R PDZbm bound SNX27 PDZ domain (PDB ID 5EM9) and AlphaFold2 multimer predicted structure of N-VARP bound to SNX27 PDZ domain

Reconstituted protein complexes	Liposome	Cargo	Imaging condition	Number of tubules counted (n)	Diameter (nm)
SNX27	PI(3)*P*	PDZbm	Negative Stain	20	38.0 ± 5.0
SNX27 + Retromer	PI(3)*P*	PDZbm	Negative Stain	50	80.0 ± 6.0
SNX27 + Retromer	Folch I	PDZbm	Negative Stain	No tubules detected	N/A
SNX2/SNX6	Folch I	None	Negative Stain	50	55.0 ± 6.0
SNX2/SNX6	Folch I	CI-MPR	Negative Stain	50	53.0 ± 5.0
SNX2/SNX6	PI(3)*P*	CI-MPR	Negative Stain	No tubules detected	N/A
SNX2/SNX6 + SNX27	Folch I	CI-MPR / PDZbm	Negative Stain	50	58.0 ± 5.5
SNX2/SNX6 + Retromer	Folch I	CI-MPR	Negative Stain	Tubules rarely detected (5)	50.0 ± 5.0
SNX2/SNX6 + SNX27 + Retromer	Folch I	CI-MPR / PDZbm	Negative Stain	50	55.0 ± 4.0
SNX2/SNX6 + SNX27	PI(3)*P*	CI-MPR / PDZbm	Negative Stain	Tubules rarely detected	N/A
SNX2/SNX6 + Retromer	PI(3)*P*	CI-MPR	Negative Stain	Tubules rarely detected	N/A
SNX2/SNX6 + SNX27 + Retromer	PI(3)*P*	CI-MPR / PDZbm	Negative Stain	50	53.0 ± 5.2
SNX2/SNX6 + SNX27 + Retromer + VARP	PI(3)*P*	PDZbm	Negative Stain	50	69.0 ± 3.5

table S3: Assessment of membrane tubule diameter via negative-stain Electron Microscopy (EM) induced by various sub-complexes in the presence of physiological lipid and cargo

**Protein**	**Peptide**	**K_d (μM)**	**ΔH (kJ/mol)**	**ΔS (J/mol-K)**	**TΔS (kJ/mol)**	**ΔG (kJ/mol)**	**n**
SNX27 PDZ (25 μM)	5-HT4(a)R-pS^{-5} (500 μM)	1.1 ± 0.2	-63.6 ± 0.5	- 99.2 ± 1.3	- 29.5 ± 1.1	- 34.6 ± 1.4	1.1 ± 0.1
Pre-incubated SNX27 PDZ and N-VARP (25 μM)	5-HT4(a)R-pS^{-5} (500 μM)	N.B.	N.B.	N.B.	N.B.	N.B.	N.B.

table S4. ITC binding data

Construct	Forward Primer (5' – 3')	Reverse Primer (5' – 3')
N-VARP E98A mutant	CTGTTCGAGGCAACCTTTTAC	GTAAAAGGTTGCCTCGAACAG
N-VARP T99A mutant	TTCGAGGAAGCCTTTTACAAC	GTTGTAAAAGGCTTCCTCGAA
N-VARP F96A/F100A mutant	ATTCTGGCCGAGGAAACCGCTTACAAC	GTTGTAAGCGGTTTCCTCGGCCAGAAT
N-VARP F96A/E98A/F100A mutant	ATTCTGGCCGAGGCAACCGCTTACAAC	GTTGTAAGCGGTTGCCTCGGCCAGAAT

table S5: Oligonucleotides (Mutagenesis Primers) used in this study
